# An Engineered Rare Codon Device for Optimization of Metabolic Pathways

**DOI:** 10.1038/srep20608

**Published:** 2016-02-08

**Authors:** You Wang, Chunying Li, Md. Rezaul Islam Khan, Yushu Wang, Yunfeng Ruan, Bin Zhao, Bo Zhang, Xiaopan Ma, Kaisi Zhang, Xiwen Zhao, Guanhao Ye, Xizhi Guo, Guoyin Feng, Lin He, Gang Ma

**Affiliations:** 1 Bio-X institutes, Key Laboratory for the Genetics of Developmental and Neuropsychiatric Disorders (Ministry of Education), Shanghai Jiao Tong University, Shanghai 200240, PR China; 2 2011 SJTU-BioX-Shanghai Team for The International Genetically Engineered Machine Competition (iGEM), Shanghai Jiao Tong University, Shanghai, PR China; 3 Shanghai genome Pilot Institutes for Genomics and Human Health, Shanghai, 200030, China

## Abstract

Rare codons generally arrest translation due to rarity of their cognate tRNAs. This property of rare codons can be utilized to regulate protein expression. In this study, a linear relationship was found between expression levels of genes and copy numbers of rare codons inserted within them. Based on this discovery, we constructed a molecular device in *Escherichia coli* using the rare codon AGG, its cognate tRNA (tRNA^Arg^ (CCU)), modified tRNA^Asp^ (GUC → CCU), and truncated aspartyl-tRNA synthetase (TDRS) to switch the expression of reporter genes on or off as well as to precisely regulate their expression to various intermediate levels. To underscore the applicability of our work, we used the rare codon device to alter the expression levels of four genes of the fatty acid synthesis II (FASII) pathway (i.e. fabZ, fabG, fabI, and tesA’) in *E. coli* to optimize steady-state kinetics, which produced nearly two-fold increase in fatty acid yield. Thus, the proposed method has potential applications in regulating target protein expression at desired levels and optimizing metabolic pathways by precisely tuning *in vivo* molar ratio of relevant enzymes.

Codon bias of rare/low usage codons has been found in several organisms, including *Escherichia coli (E. coli)*, *Saccharomyces cerevisiae*^1^,^2^,^3^, *Drosophila*^4^, *Caenorhabditis elegans*^5^, *Synechococcus*, and *Prochlorococcus*^6^. Codon bias has been reported to be correlated with the relative abundance of specific tRNAs^7^,^8^,^9^. As the rarest codons, AGG, AGA, CUA, AUA, CGA, and CCC of *E. coli* are essential in regulating the expression of different endogenous proteins^10^,^11^. The relative positions of these rare codons in genes correlate with levels of suppression of protein expression in *E. coli*^12^,^13^,^14^,^15^,^16^,^17^. This suppression, which occurs mostly at the translational level, is due to shortage of cognate tRNAs corresponding to these rare codons. Therefore, co-expression or overexpression of these cognate tRNAs is believed to overcome the impediment to protein expression caused by rare codons^18^. Modified aminoacyl-tRNA synthetases (aaRS) are often paired with the cognate mutant tRNA, which is mutated at its anticodon arm to permit interaction with the target codon without affecting amino acid attachment, to recognize codons in various applications. This is especially useful for codon expansion, which is frequently utilized to incorporate unusual amino acids into protein sequences^19^,^20^,^21^,^22^. However, little is known about how protein expression is precisely regulated by rare codons and mutant tRNAs.

Controlling transcription and translation using synthetic genetic tools is pivotal in engineered biosystems for improving host capacity to produce value added products. Although numerous regulation tools have been developed, a few of them have simply been applied for tuning tandem enzymes, in which concurrent action of several enzymes work together and sequentially in the metabolic pathway of a biological system^23^. To achieve this aim, we formulated and assembled molecular devices utilizing the rare codon AGG and cognate tRNA^Arg^ (CCU) of AGG, mutated tRNA of aspartic acid (tRNA^Asp^ (GUC → CCU)) and truncated aspartyl-tRNA synthetase (TDRS) of *E. coli*, and mutated tRNA of *N*-formylnethionine (tRNA^fMet^ (CAT → TCG)) and truncated methionyl-tRNA synthetase (MetRS) to regulate and tune protein expression. To inhibit the expression (off-state) of target genes, devices were constructed and tested by inserting rare codons immediately after the start codon of the reporter genes *red fluorescent protein* (*RFP*) and *luciferase* (*luc*). To switch “on” the off-state gene inserted with rare codons, we overexpressed the cognate tRNA of the rare codon. Under constant expression of the cognate tRNA, the level of protein expression was found to be correlated with number of copies of rare codon and we used this principle to fine-tune the expression level of a target reporter gene precisely using different number of copies of rare codons. Surprisingly, we found that the ratio of inserted rare codon number in the target gene and its expression was constant both under a strong and weak promoter indicating protein production was correlated with the rare codon number only at constant cognate tRNA level.

In practical applications of rare codon devices, an arginine tag is translated from the rare codon AGG, which may affect the function of target proteins. To overcome this potential disadvantage, we designed a helper system to remove the tag via intein self-slicing *in vivo* after translation of target proteins. Intein is a family of proteins that catalyzes splicing in the cis or trans position^24^. The well-studied intein from *DnaE* of *Synechocystis* spp. PCC6803 was selected and constructed using existing rare codon devices^25^,^26^. Our results showed that the helper system successfully removed the tag while retaining the properties of rare codon devices.

Finally, we used this instrument to optimize the fatty acid metabolic pathway in *E. coli.* Fatty acid biosynthesis in *E. coli* is catalyzed by an enzyme system (FASII) consisting of nine distinct proteins: FabA, FabB, FabD, FabF, FabG, FabH, FabI, FabZ, and ACP (Fig. S1). These proteins cooperatively convert one equivalent of acetyl-CoA and 6–8 equivalents of malonyl-CoA into C14–C18 acyl-ACP species. Periplasmic thioesterase, TesA, is capable of releasing free fatty acids via hydrolysis of acyl-ACP species. According to a previous steady-state kinetic analysis of FASII, an optimal molar ratio of 1:1:1:1:1:1:10:10:30:30 FabA:FabB:FabD:FabF:FabG:FabH:FabI:FabZ:holo–ACP:TesA is necessary to achieve a maximum rate (100 μM/min) of free fatty acid synthesis in *E. coli*^27^. Titration-based *in vitro* kinetic analysis and other biochemical studies suggest that the appropriate molar ratio of fabG, fabI, fabZ, and tesA is rate-limiting in fatty acid over-production in *E. coli*^27^,^28^,^29^. Guided by these works, we selected the genes fabG, fabI, fabZ, and tesA’ (tesA’ is tesA mutant without the signal sequence peptide that redirects it to localize in the cytoplasm to increase the accessibility of substrates to the enzyme’s active site)^29^ to optimize the FASII pathway. Since we found that the number of inserted rare codons is related to the expression level of the gene, we inserted different number of copies of rare codon into fabG, fabI, fabZ, and tesA genes, aiming to get an approximate expression ratio of 1:10:10:30 (fabG:fabI:fabZ:tesA) targeting over-production of free fatty acids. Collectively, our results provide novel insight into the potential applications of these codon devices in controlling and fine-tuning the expression of target proteins and regulating proteins in the metabolic pathway.

## Results

### The expression level of target genes controlled by rare codon devices depends on rare codon number

Insertion of rare codons after the start codon of a gene suppresses its expression, whereas overexpression of its cognate tRNA restores this expression. Rare-codon devices were designed in this study based on this premise. To test this idea, the rare codon of *E. coli*, AGG, was inserted immediately downstream of the start codon of the reporter gene *RFP* to silence its expression. The gene of the cognate tRNA of AGG, tRNA^Arg^ (CCU), was assembled under an IPTG-inducible promoter, *trc*, to switch the already silenced gene expression on (Fig. S2A). Cells harboring the constructed plasmid with only *RFP* under the *T7* promoter, pET-*T7*-RFP, were found to express RFP normally. This expression, however, ceased in cells harboring the pET-*T7*-RFP-6AGG plasmid, in which 6 AGG codons were inserted immediately after the start codon (Fig. S2B). Gene expression was restored in cells containing both pET-*T7*-RFP-6AGG and pACYC-*trc*-argW which indicates that overexpression of the tRNA of a rare codon is sufficient to permit protein expression (Fig. S2B).

**Figure 1:**
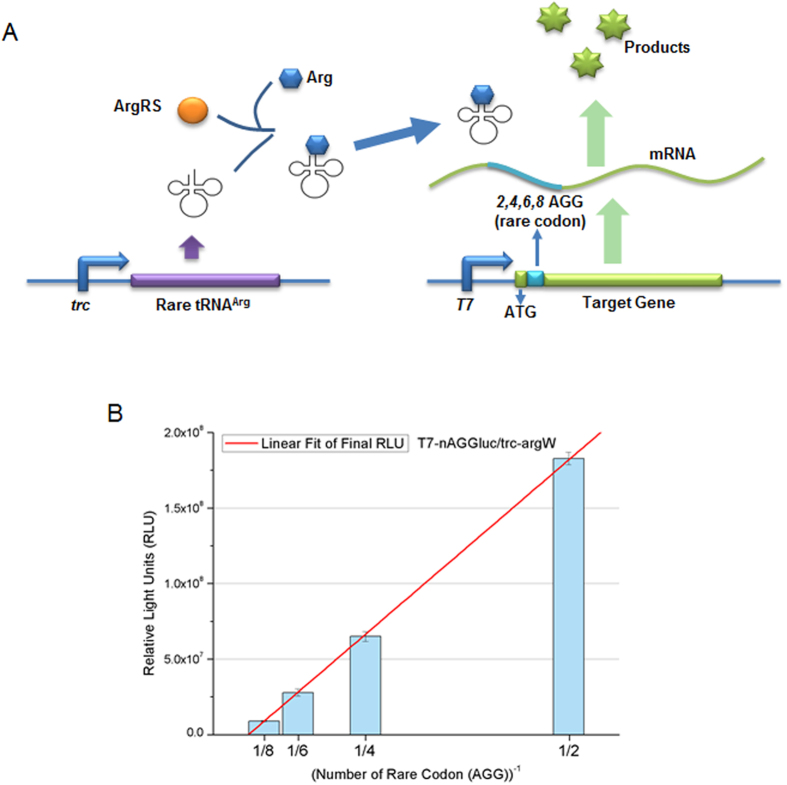
Regulation of luciferase expression by rare AGG codon insertion. (**A**) Constructs used to generate rare codon devices. Various numbers of rare AGG codons (cyan) were inserted immediately downstream of the start codon of luciferase gene (green) under the influence of the *T7* promoter (blue). The cognate tRNA^Arg^ (CCU) gene (purple) was influenced by the *trc* promoter (blue). The expression level of the target protein was controlled by the number of rare codon (AGG) insertions. **(B)** Regulation of luciferase expression using the constructs shown in Fig. 1A. The results observed were fitted to Eq. (1) and are depicted in Fig. 1B (red line).

We analyzed the effect of the number of inserted rare codons on gene expression. Constructs containing the *luc* reporter gene were assembled, each with a different number of AGG copies inserted, under a strong and inducible promoter, *T7* (Fig. 1A). Cells harboring the devices pET-*T7*-luc-AGG and pACYC-*trc*-argW showed distinguishable expression levels of luciferase at different time intervals after induction by IPTG (Fig. 1B and S2C–S2I). Interestingly, the expression levels of *luc* genes corresponded to the number of inserted rare codons even when the cognate tRNA was overexpressed. Higher copy number of the inserted rare codons decreased the expression level of luciferase, as determined by luciferase activity assay and SDS-PAGE (Figs 1B,S3A, and S3B). Moreover, protein expression could be linearly fitted to an equation [Eq. (1)] as a function of rare codon number^−1^. Based on this function, we obtained the relative ratio of gene expression levels for different numbers of AGGs (Table 1). Thus, luciferase levels can be precisely controlled by varying the copy number of inserted rare codons.

### The relationship between protein expression and rare codon number is constant

**Figure 2:**
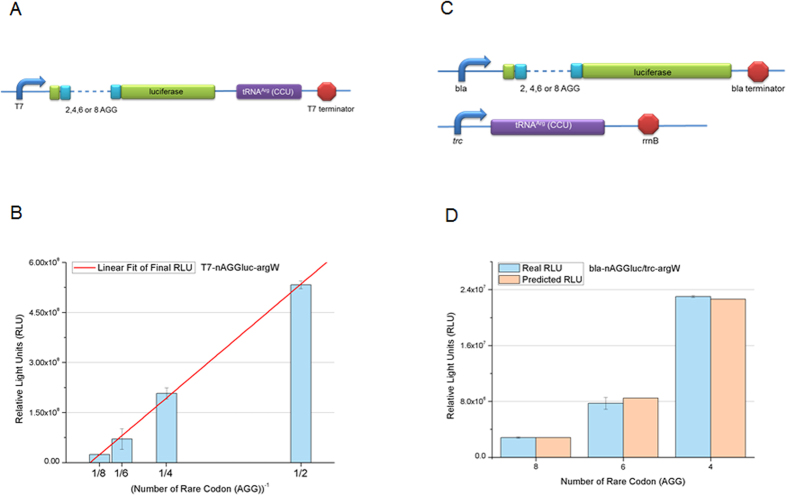
Relationship between the number of AGG insertions and protein expression in different regulation systems. (**A**) Constructs of rare codon devices with co-transcribed cognate tRNA^Arg^ (CCU) and the luciferase gene (green) with different numbers of AGG codons (cyan) under the *T7* operon. (**B**) Regulation of luciferase expression using constructs shown in Fig. 2A. (**C**) Constructs of rare codon devices with the luciferase gene transcribed by the weak *bla* promoter. (**D**) Comparison between the real (cyan) and predicted (peach tint) results of luciferase expression regulated by the rare codon devices. Luciferase expression was predicted based on relative light units of luciferase with eight AGG codons and their ratios in Table 1.

To determine whether the ratio of gene expression levels for different numbers of AGGs is stable so that the devices can be used universally, further experiments were conducted. First, the cognate tRNA was integrated into the same operon as the target gene (Fig. 2A) to determine the effect of tRNA location on reporter gene expression. The device displayed linear and stable expression ratios (Fig. 2B, Table 1). Next, gene expression was measured under different promoters/rbs (ribosome binding site) to determine whether expression levels could be predicted based on the number of rare codons and regardless of promoter strength and difference in *rbs* (Fig. 2C). To do this, we considered luciferase expression with 8 AGG insertions as base to predict (Table 1) what the level of expression of 4 or 6 AGG would be under the weak and constitutive *bla* promoter, which is different than the promoter used for 8 AGG. When the luciferase gene was expressed with 4, 6 and 8 AGG insertions under the *bla* promoter, we found that the expression level was surprisingly similar to the predicted level (Fig. 2D). This suggests that prediction of target protein expression may be possible using the appropriate rare codon switch without the difficult work of experimentally determining the appropriate rare codon copy number. Thus, the relationship between protein expression and rare codon number is very consistent regardless of tRNA location or expression and promoter/rbs strength. Moreover, the target protein expression level can be easily predicted when using a rare codon switch, as one can choose the appropriate number of inserted rare codons to achieve the required expression level.

### Truncated aminoacyl-tRNA synthetase increases the diversity of rare codon devices

**Figure 3:**
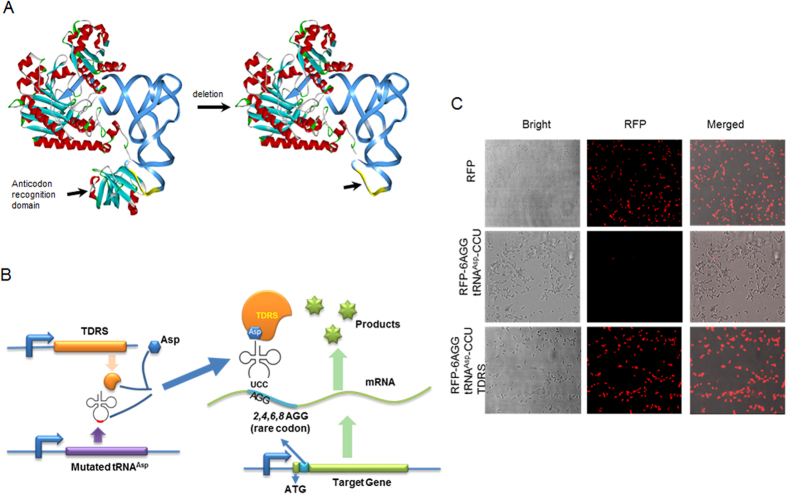
Rare codon devices obtained by modifying aminoacyl-tRNA synthetase and the cognate tRNA. (**A**) Modification of *E. coli* aspartyl-tRNA synthetase. The anticodon recognition domain of aspartyl-tRNA synthetase was truncated based on available structural data (PDB ID: 1C0A). (**B**) The scheme of the rare codon device with TDRS. Various numbers of rare AGG codons (cyan) were inserted immediately downstream of the start codon of target gene, RFP or luciferase gene (green) under the influence of the *T7* promoter (blue). The TDRS (orange) and the mutated tRNAAsp (GUC → CCU) gene (purple) were influenced by the *T7* promoter (blue). The expression level of the target protein was controlled by the number of rare codon (AGG) insertions and the AGG codon here was translated to Asp. (**C**) Cells transformed with plasmids containing *RFP* without rare AGG codons, mutated tRNA^Asp^ (GUC → CCU), and TDRS normally expressed RFP (upper row). RFP expression nearly ceased upon insertion of 6 AGGs immediately after the start codon of the *rfp* gene, even with co-expression of tRNA^Asp^ (GUC → CCU) (middle row). RFP expression was restored in cells co-transformed with tRNA^Asp^ (GUC → CCU) and TDRS (bottom row).

From a mathematical standpoint, amino acids and codons can be combined in numerous permutations. However, natural amino acid-codon pairs impose limits on the possibilities for our rare codon device. Although cognate tRNA overexpression restored the expression of genes with rare codon insertion, we were interested in diversifing our devices by using other modified tRNAs. To do this, we truncated aminoacyl-tRNA synthetase and mutated the anticodon of the cognate tRNA (to allow base pairing with AGG or other inserted rare codons) with the expectation that the truncated aminoacyl synthetase will charge the mutated tRNA which will eventually pair with the targeted rare codon and overcome the impediment of translation. The truncated enzyme was constructed by deleting the anticodon recognition domain of the enzyme so that it could recognize and charge the mutated tRNA. The charged tRNA may then be used to translate the AGG codons. Aspartyl-tRNA synthetase and its cognate tRNA^Asp^ from *E. coli* were chosen to construct the truncated Aspartyl-tRNA synthetase (TDRS) (Fig. 3A) and tRNA^Asp^ (GUC → CCU), respectively. TDRS had 106 aa deleted from its anticodon recognition domain, and the anticodon of tRNA^Asp^ was mutated from GUC to CCU (Fig. 3B). TDRS and tRNA^Asp^ (GUC → CCU) were respectively controlled by the *T7* promoter and its native and constituent promoter. *RFP* (with 6 AGG insertion) and *luc* (with 4 AGG insertion) were used as reporter genes to test this system. RFP expression ceased in the absence of TDRS expression but was restored upon TDRS induction (Fig. 3C). Consequently, the tRNA^Asp^ (GUC → CCU) that recognizes the AGG codon was charged with aspartate by TDRS, thereby restoring the expression of RFP. A similar result was observed when luciferase (with 4AGG insertion) was used as the reporter gene (Fig. S4). To test this strategy further, the anticodon recognition domain (1–374 aa) of methionyl-tRNA synthetase (MetRS) was deleted, and the anticodon of its corresponding tRNA^fMet^ (fMet, formyl-methionine) was mutated (CAT → TCG) to recognize the rare codon CGA. The kanamycin resistance gene was chosen as a reporter gene, and the start codon ATG was changed to CGA to test the function of truncated MetRS (Fig. S5A). No bacteria colonies formed on the kanamycin plate in the absence of truncated MetRS expression; by contrast, a large number of colonies emerged when truncated MetRS was expressed (Fig. S5B). Thus, truncated MetRS and its corresponding mutated tRNA^fMet^ do, in fact, initiate the expression of the reporter gene with the abnormal start codon CGA, which confirms that any other aaRS can be modified similarly to recognize the rare codon. Our findings indicate that the same strategy can be applied to other aminoacyl-tRNA synthetase and tRNA pairs.

### Addition of the intein system removes rare codon tags

**Figure 4:**
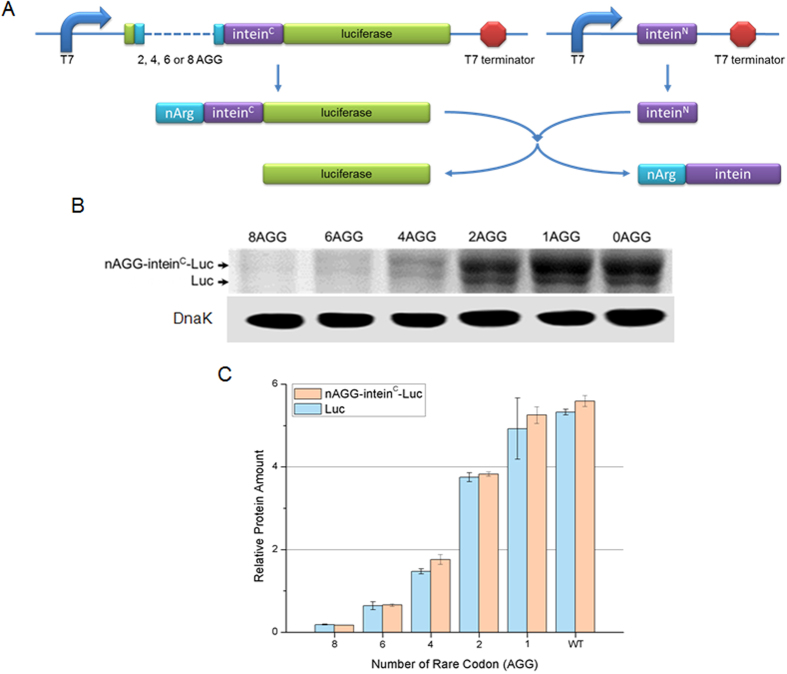
Removal of the arginine tag via an additional intein splicing system. (**A**) Schematic view of the intein splicing system. Of the two parts of the intein system, intein^C^ was attached to the rare codon while intein^N^ was expressed independently. After expression, Arg-tagged intein^C^ and intein^N^ splice and joined together leaving luciferase free of Arg tag via trans-splicing. (**B**) SDS-PAGE of lysate from cells harboring a rare codon device and the intein splicing system depicted in Fig. 4A. The upper bands (nAGG-intein^C^-Luc) represent luciferase with various numbers of arginines and intein^C^ at the N terminus. The middle bands indicate only luciferase proteins that were free from the Arg tag generated from nAGG-intein^C^-Luc and intein^N^ splicing. The lower bands are western blots of DnaK protein, which was chosen as the internal control (DnaK monoclonal antibody, ADI-SPA-880-D, Enzo Life Sciences) (**C**) The bands in Fig. 4B were quantified in terms of relative protein amount using Quantity One® software.

One potential disadvantage of the rare codon device is that rare codons responsible for regulation are translated, resulting in a ‘rare codon’ tag at the N terminus of the target protein. Although we have not found any functional disability in the rare codon inserted protein we tested but we fear that it may happen in the case of many proteins. Thus, removing the tag might increase the ability of this device for universal application. To remove this tag, we developed a helper system, which we call the “intein system”. In the intein system, the two fragments of a protein, named C-terminal intein (intein^C^) and N-terminal intein (intein^N^), get joined together by ligating fragments attached to them via self-splicing. The well-characterized intein system has previously been used to remove such tags as posttranslational modification *in vivo*^25^. Tandem rare codons and shorter C-terminal intein (intein^C^) were added to the N terminus of luciferase during cloning, and N-terminal intein (intein^N^) was cloned into another vector as a helper that constantly exists in the cell (Fig. 4A). By co-expressing luciferase with the rare codon tag and intein^C^, and intein^N^ in cells, rare codon tag and intein^C^ will be removed by self-splicing, resulting in a luciferase reporter without a tag and a mature intein peptide with a rare codon tag (Fig. 4A).

To test this design, the widely used DnaE intein^C^ from cyanobacteria (PCC6803) was cloned between luciferase and tandem rare codons, and intein^N^ was cloned in pRSFDuet vector. Lysis of the cell containing two vectors showed two overexpressed protein bands: luciferase with a rare codon tag (arginine tag) and intein^C^ as well as luciferase without a tag (Fig. 4B). Quantitative analysis of these bands demonstrated that the self-splicing rate was about 50% and constant among different constructs with various numbers of AGG insertion, thereby resulting in a profile similar to the expression of luciferase regulated by the rare codon device without the intein system (Figs 4C and S3B). Applying the additional intein system, the rare codon tag was successfully removed without any detriment to the properties of the rare codon device.

### Rare codon devices modulate fatty acid synthesis in *E. coli*

**Figure 5:**
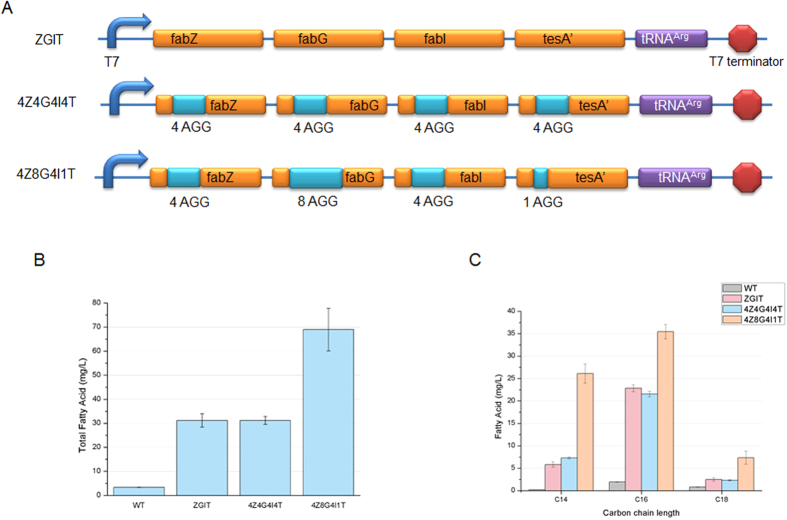
Modification of the fatty acid synthesis pathway in *E. coli* via using rare codon devices to regulate expressions of fabZ, fabG, fabI and tesA’. (**A**) Constructs of four modified genes in the fatty acid synthetic pathway (4Z8G4I1T) and controls (4Z4G4I4T and ZGIT). The numbers represent the number of rare codons (AGG) inserted immediately after the start codon. (**B**) Comparison of total fatty acids produced in *E. coli* containing the modified construct (4Z8G4I1T) and controls (4Z4G4I4T, ZGIT and WT) shown in Fig. 5A. (**C**) Yields of fatty acids with 14 (C14), 16 (C16) and 18 (C18) carbons were determined and compared among WT *E. coli* and the strains harboring the plasmids mentioned in 5A.

To demonstrate the applicability and efficiency of our rare codon devices, we tested these devices for their ability to optimize the fatty acid synthesis pathway in *E. coli*. As previously determined, the ratio of fabZ, fabG, fabI, and tesA’ must be 10:1:10:30 to achieve optimal activity of the FASII pathway. Based on this information, we constructed a vector with genes containing different numbers of rare codon AGGs in order to produce these enzymes in the aforementioned molar ratio and tune the FASII pathway. First, we developed the *E. coli* strain ZGIT (Fig. 5A) with normal overexpression of these four essential genes. Based on our previous results (Table 1), we hypothesized that a ratio such as 1:10:30 may be successfully achieved by inserting rare codons in the proportion of 8:4:1. Accordingly, *E. coli* strain 4Z8G4I1T (Fig. 5A) was constructed with the same four genes but with different numbers of AGG codons (4 AGG within fabZ, 8 AGG within fabG, 4 AGG within fabI, and 1 AGG within tesA’) inserted right after the start codon to obtain expressions at a molar ratio of approximately 10:1:10:30 (fabZ, fabG, fabI, tesA’). The *E. coli* strain 4Z4G4I4T (Fig. 5A) was also constructed with the same four genes but with insertion of 4 AGG to ensure down-regulation and equal proportions of expression; the resulting strain further served as a control. According to GC-MS measurements of extracellular fatty acid, the main fatty acid produced in WT and other engineered strains was hexadecanoic acid (C16:0, C16:1), which was accompanied by small amounts of tetradecanoic acid (C14:0) and octadecanoic acid (C18:0, C18:1). As expected, the amount of total fatty acids produced in the strain 4Z8G4I1T was significantly higher than that produced by the control strains ZGIT, 4Z4G4I4T and WT (Fig. 5B,C). The *E. coli* strain 4Z8G4I1T showed the highest production of total fatty acids (68.96 mg·L^−1^) (Fig. 5B), which was around two folds higher than those produced by the two other strains, ZGIT (37.17 mg·L^−1^) and 4Z4G4I4T (37.16 mg·L^−1^). The total fatty acids production in ZGIT and 4Z4G4I4T strain was also found higher than WT. The quantities of individual constituents in the final product of the strain 4Z8G4I1T, namely C14, C16, and C18, increased by 5-fold, 1.5-fold, and 3-fold, respectively, compared to the corresponding products obtained from the two other strains (Figs 5C and S6). When we compared the individual fatty acids C14, C16 and C18 between WT and other engineered strain, it was found that all other strains were superior in production than WT. However, the composition of total fatty acids did not vary between engineered strain and WT suggesting that maintaining the molar ratio of key enzyme using rare codon did not interfere with other normal function of the pathway.

## Discussion

Numerous molecular tools have been developed to control biosystems for different purposes. Transcription and translation are the most common targets for regulation in pathway optimization to produce value added products from microbial hosts. Unlike transcription tools that produce tight but rough regulation, translation tools such as modified RBS^30^, riboswitch^31^, and translation initial elements^32^ are generally designed to precisely control individual products in cells. Beyond manipulation of individual targets, the ratio of tandem enzymes in the pathway is a crucial parameter for optimizing metabolic products, such as those in the fatty acid biosynthetic pathway, which was optimized in this work. In order to achieve a stable and constant ratio of a set of enzymes, we focused on building devices using rare codons and tRNAs to control and tailor expression of various genes at the translation stage.

Previous studies^11^,^33^ have suggested that impediment of protein synthesis of genes with tandem insertion of rare codons can be attributed to deficient supply of the cognate tRNAs of these rare codons. Moreover, Rosenberg *et al.* found that insertion of tandem copies of five other high-usage codons, i.e. GGA (Gly) and GAG (Glu) instead of the rare AGG (Arg), did not have significant effects on translation significantly^11^. This supports the hypothesis that rare codon usage is linearly related to impediment to translation. Collectively, these findings indicate that tRNAs are strong modulators of translation alteration. Moreover, tRNA^Arg^ (CCU) can potentially become sequestered in a peptidyl-tRNA^Arg^ (CCU)-ribosome triple complex that can halt translation if the copy number of the rare codon AGG in the mRNA is higher than the number of tRNA^Arg^ (CCU) in the system^34^. Therefore, an abundance of tRNA^Arg^ (CCU) or recognition of the rare codon by an alternative tRNA can remove this impediment to protein translation. In the present work, we showed that switching devices composed of the reporter gene *rfp* with 6 tandem AGGs immediately downstream of the start codon could stop RFP expression almost completely whereas overexpression of the cognate tRNA of AGG and tRNA^Arg^ (CCU) restored RFP expression. Thus, rare codons and their corresponding tRNAs can be developed as modular switches to turn protein expression on or off (Fig. S2A,B).

The abundance of tRNA^Arg^ (CCU) may be utilized in controlling the level of expression of a gene containing AGG insertions. tRNA^Arg^ (CCU) was overexpressed under separate operons and co-expressed in the same operon as a reporter gene to verify the above hypothesis. Increasing the number of copies of the inserted rare codons clearly decreased the expression level of the luciferase reporter (Fig. 1B). Moreover, the relationship between protein expression and rare codon number^−1^ is linear, and the relative ratio derived from the linear functions is constant regardless of tRNA location or expression and promoter/rbs strength. Furthermore, the expression levels of target genes with different rare codon numbers can be predicted easily using this constant ratio. The mechanism of variations in expression level caused by different numbers of rare codon insertion remains unclear. We believe that limitation of the amount of arginyl-tRNA available by ppGpp (guanosine tetraphosphate), which is involved in regulating translation-related processes, may be responsible for this phenomenon, although the tRNA^Arg^ (CCU) was overexpressed in our codon-switch system^35^. Thus, the phenomenon of variation in expression level caused by different numbers of rare codons was exploited in the rare-codon switch devices to precisely control target genes of interest by inserting the appropriate number of rare codons.

Our results clearly demonstrate the potential applications of rare codon devices. However, these devices are limited by natural amino acid-codon pairs. To increase the variety of rare codon devices, we designed truncated aminoacyl-tRNA synthetase and mutated the anticodon of the cognate tRNA to base pair with AGG or other codons and break the “one codon, one amino acid” rule. To test this strategy, we modified *E. coli* aspartyl-tRNA synthetase and the cognate tRNA, which resulted in truncated aspartyl-tRNA synthetase and mutated tRNA^Asp^ (GAC → CCU). The anticodon sequence of tRNA^Asp^ (GUC) was changed to tRNA^Asp^ (CCU) by point mutation to verify its ability to recognize the AGG codon. Aspartyl-tRNA synthetase is responsible for charging the tRNA^Asp^ (GUC), and this charging is specified by the anticodon sequence of tRNA^Asp^, GUC. The anticodon-recognizing domain of 106 amino acids (318 bp), based on the crystal structure of aspartyl-tRNA^Asp^ synthetase in *E. coli*, as revealed by Eiler *et al.*^36^, was deleted from the N-terminal fragment of aspartyl-tRNA synthetase to charge the mutated tRNA^Asp^ (GUC → CCU). Thus, the truncated aspartyl-tRNA synthetase (TDRS) lost its specificity for tRNA^Asp^ (GUC), the original substrate, and gained the ability to charge tRNA^Asp^ (CCU) without specificity. Co-expression of both tRNA^Asp^ (GUC → CCU) and TDRS rescued the arrested protein expression of the 6 AGG-inserted *rfp* (Fig. 3B,C), indicating that this pair is a strong modulator for charging aspartate with the rare AGG codon. Analysis of the complex structures of some other tRNAs and the corresponding aaRS binding pairs showed that some of these molecules have structures similar to that of the binding pair of tRNA^Asp^ and aspartyl-tRNA synthetase, including arginyl-tRNA synthetase, cysteinyl-tRNA synthetase, glutaminyl-tRNA synthetase, glutamyl-trna synthetase, threonyl-tRNA synthetase, tryptophanyl-tRNA synthetase, tyrosyl-tRNA synthetase, and methionyl-tRNA synthetase (Fig. S7, Discovery Studio Visualizer 2.5, Accelrys). The anticodon-recognizing domains are independent of the other parts of these synthetases; thus, deletion of an anticodon recognition domain should have no significant effect on the structure and activity of these synthetases. Truncation of synthetases was explored by purposefully adopting the same methods described for TDRS and truncated MetRS. Hypothetically, TDRS should also charge other tRNAs and affect proteins as TDRS has lost its specificity. However, we did not see any phenotypic changes in cells expressing TDRS. This may be so because other normal tRNAs synthetase that have unaltered anticodon-detecting domain maintain highly specific charge reactions for their respective tRNAs. Thus, there are no more other tRNAs available, compared to tRNA^Asp^ (GUC → CCU), to be charged.

Our experiments showed that *rfp* and *luciferase* genes inserted with different numbers of rare codon AGG, which encodes the polar amino acid arginine, maintained their biological activities. However, normal activity of other target proteins with the rare codon tag cannot be guaranteed. To overcome this potential disadvantage of rare codon devices, we designed an additional helper system to remove the rare codon tag as a post-translational modification. By applying intein^C^ between the rare codon tag and luciferase, the rare codon tag was removed via spontaneous intein self-splicing. The properties of intein self-splicing have been highly developed and widely used in several areas^24^. Since we chose the well-studied DnaE intein, 50% of the rare codon tags were removed by intein self-splicing, which is consistent with previous data^37^. By applying more efficient natural or engineered inteins, this number can be expected to reach nearly 100%^26^. Thus, based on our results and those of previous studies, potential problems caused by the rare codon tag can be solved by the use of an intein helper system.

To demonstrate the applicability and efficiency of our proposed devices, we tested the rare codon device for its ability to control the metabolic pathway of *E. coli*. Considering its well-studied impact on biofuel production, the *E. coli*’s FASII pathway (Fig. S1) was selected to test the regulation efficiency of our devices. Steady-state kinetic analysis indicated that different combinations of a molar ratio of four rate-limiting enzymes of FASII, i.e., fabZ, fabG, fabI, tesA’ (10:1:10:30), have remarkable influences on fatty acid over-production^27^. As seen in Fig. 5B, total fatty acid (including C14, C16 and C18) production increased in the ZGIT over-expression strain. To obtain a downregulating and equal molar ratio of the four enzymes, we inserted four AGGs immediately downstream of the start codon of each of the four genes. As expected, fatty acid production in *E. coli* 4Z4G4I4T was nearly equal to that in ZGIT (Fig. 5B). Since we used the strong *T7* promoter to drive expression of these genes, the enzymes productions must exceed the amount needed in ells. Considering that the enzymes are superfluous relative to the small amount of substrate, the product yield must depend only on the ratio of the enzymes, which implies that equimolar ratios of these four enzymes (1:1:1:1 in both ZGIT and 4Z4G4I4T) should lead to the production of equivalent quantities of fatty acids. Maximum levels of fatty acid production were observed in the 4Z8G4I1T strain (in which four enzymes were expressed at a molar ratio of approximately 10:1:10:30), with nearly two-fold higher yield compared to those of the ZGIT and 4Z4G4I4T strains (Fig. 5B). These results reinforce our earlier conclusions regarding the regulatory capacity of the rare codon device and demonstrate its applicability and efficiency in controlling metabolic pathways. Metabolic flux balance is essential in the field of microbial metabolic engineering, and most reactions that take place in organisms are sensitive to variation of even only a few enzymes. Therefore, accommodation of intracellular carbon fluxes through a specific metabolic pathway presents a rapid and efficient method for improving the value added to commercial products. With the help of the rare codon switch device, one can conveniently adjust expression levels of several genes to optimal levels, which greatly aids optimization and reconstruction efforts to produce rigorous and effective metabolic networks in living organisms.

Over the last few decades, researchers have revealed the mystery of rare codons, their role in controlling cellular behavior, and their mechanism of action. Engineered or orthogonal tRNA/aaRS pairs are concomitantly used in expanding codons to insert unnatural amino acids in a site-specific manner. These advanced molecular approaches were exploited for the first time in the present study to build devices with high potential for regulating protein expression. The rare codon devices designed in this study can not only switch the expression of a gene of interest on and off *in vivo* but can also control its level of expression. Collectively, our results provide a robust proof-of-principle for applying the rare codon device in various molecular approaches to study gene function and to fine-tune complex metabolic pathways.

## Materials and Methods

### Strains and constructs

*E. coli* DH5α was used for cloning, while *E. coli* BL21 (DE3) were used for protein expression. We considered expression of different genes on vector rather than inserting it into the genome of *E.coli*. The vectors for the constructs included pET28a, pACYC184, pRSF-Duet1, and pTrc99b.

The *luc* gene was amplified using the primers *n*luc-NcoI-F (*n* = 2, 4, 6, 8) and luc-NdeI-R from the template *Photinus pyralis luciferase* gene (BioBrick part: BBa_I712019) obtained from the BioBrick parts registry at MIT (http://partsregistry.org/Main_Page). AGG codons (each with a different number of AGG copies: 2, 4, 6, and 8, designated as 2AGG, 4AGG, 6AGG, and 8AGG, respectively) were introduced after the second codon of the *luc* gene. Polymerase chain reaction (PCR) products were inserted into a pET28a vector using *NcoI* and *NdeI* (Fermentas) to produce pET-*T7*-2AGGluc, pET-*T7*-4AGGluc, pET-*T7*-6AGGluc, and pET-*T7*-8AGGluc constructs. These constructs were transformed into DH5α cells, and the resulting clones were inoculated into Luria broth (LB) containing kanamycin and cultured at 37 °C.

In another series of pET-bla-*n*AGGluc vectors (*n* = 4, 6, 8), the *luc* gene was constructed under the *bla* promoter from the *ampR* operon of the pUC18 vector. The a*mpR* operon, which features a sequence homologous to that of pET-28a, was amplified from the pUC18 vector using the primers ampR-F and ampR-R. The amplified *ampR* operon was inserted into pET-28a, thereby replacing a nonessential region by overlap extension PCR and resulting in the vector pET-ampR. This construct was transformed into DH5α and screened by ampicillin and kanamycin. The *luc* ORF was amplified using the primers *n*bla-luc-F (*n* = 4, 6, 8AGG) and LucAmp-R, the 5′ ends of which were complementary to the sequences flanking the *ampR* that replaced the beta-lactamase ORF (the gene in *ampR* operon) by overlap extension PCR^38^ to express the *luc* gene under the *bla* promoter. In this process, the ampicillin resistance conferred by the plasmid was eliminated. This homologous sequence was used to insert the *luc* gene into pET-ampR, and the resulting construct was named pET-bla-*n*AGGluc. The constructs obtained were transformed into DH5α cells, and the resulting clones were separately inoculated into LB containing kanamycin and ampicillin. Clones that grew on kanamycin but did not survive on ampicillin contained the correct target vector.

The *RFP* gene was obtained from the BioBrick registry at MIT (BioBrick part: BBa_I13521). The vector pET-*T7*-RFP carrying the wild type *RFP* gene was constructed by amplifying the *RFP* gene using the primers rfp-F and rfp-R. The resulting PCR product was inserted into the pET-28a vector using *NdeI* and *XhoI*. Six copies of the rare codon AGG (6AGG) were inserted after the *RFP* initial codon through PCR reactions using the primers 6rfp-F and 6rfp-R. The PCR product was then inserted into pET-28a and pACYC-*T7*-aspV (CCU) vectors (this vector will be further discussed below) using *NdeI* and *XhoI*, resulting in the vectors pET-*T7*-6AGGRFP and pACYC-*T7*-6AGGRFP-aspV (CCU). pACYC-*T7*-4AGGluc-aspV (CCU) was also constructed. The *luc* gene with a 4AGG insertion was placed into the plasmid pACYC-*T7*-aspV (CCU) in the same manner as that used in the construction of pET-*T7*-4AGGluc.

The *LacI* regulator, *trc* promoter, and *rrnB* terminator, derived from pTrc99b^39^ as a whole, were amplified and inserted into the pACYC184 vector through overlap extension PCR using the primers trc-F and trc-R. The underlined sequences of trc-F and trc-R (Table S1) are homologous to that in pACYC184; the resulting construct was called pACYC-*trc*. tRNA^Arg^ (CCU) (where CCU represents the anti-codon) and *argW* sequences were amplified from *E.coli* K-12 strain genome using the primers *argW*-F and *argW*-R, which have sequences homologous to that of the vector pACYC-*trc*. The resulting products were then inserted into pACYC-*trc* through overlap extension PCR under the control of the *trc* promoter and *rrnB* terminator. The resulting construct was named pACYC-trc-argW and used to express tRNA^Arg^ (CCU).

The *aspS* gene was transcribed as aspartyl-tRNA synthetase (aspS). The anti-codon recognition domain (containing 106 amino acids) of this gene was deleted when only the C-terminal fragment of the *aspS* gene is amplified^36^,^40^ from the genome of the *E. coli* K-12 strain using the primers TDRS-F and TDRS-R. The PCR product, which codes truncated aspS, was inserted into a pET-28a vector using *NcoI* and *NdeI* and the resulting construct was named pET-*T7*-TDRS.

The *aspV* operon, containing its original promoter and terminator, was amplified from the genome of *E. coli* K-12 using the primers *aspV*-F and *aspV*-R and then inserted into pACYC184 with its homologous sequences flanking the *CmR* operon such that the *aspV* operon replaced the *CmR* operon in pACYC184, resulting in the vector pACYC-aspV. The *T7* operon, containing both the *T7* promoter and *T7* terminator, from pET-28a was amplified using the primers *T7*-F and *T7*-R and then inserted into pACYC-aspV to replace a nonessential region by overlap extension PCR, resulting in the pACYC-*T7*-aspV vector. The anti-codon of the tRNA was changed from GUC to CCU or CUA to match the codons AGG through point mutations using the primer pair *aspV*-CCU-F/*aspV*-CCU-R. The resulting constructs were named pACYC-*T7*-aspV (CCU).

The *argW* gene^41^ was amplified from the *E. coli* K-12 strain genome using the primers FB-argW-F and FB-argW-R. The PCR product was inserted into pET-*T7*-2AGGluc after the *luc* gene via the control of the *T7* promoter. This construct was called pET-2AGGluc-argW. pET-*T7*-4AGGluc-argW, pET-*T7*-6AGGluc-argW, and pET-*T7*-8AGGluc-argW were constructed using the same procedure.

The plasmids in the intein system were derived from pET-*T7*-nAGGluc-argW. The genes of N-terminal and C-terminal inteins were amplified from *DnaE* of *Synechocystis* spp. PCC6803. The C-terminal intein gene was inserted into pET-*T7*-nAGGluc-argW between nAGG and the luciferase gene by PCR cloning. The N-terminal intein gene was inserted into pRSF-Duet1 as the helper plasmid to supply sufficient N-terminal inteins for self-splicing.

In the fatty acid biosynthesis experiment, three Fab genes (i.e., FabZ, FabG, and FabI), TesA’, and the *argW* gene were amplified from the *E. coli* K-12 strain genome using primers listed in Table S1. These genes were inserted one by one between the *NcoI* and *XhoI* sites of pET28a vector to produce pET-*T7*-ZGIT-argW (hereafter called ZGIT), pET-*T7*-4Z4G4I4T-argW (hereafter called 4Z4G4I4T), and pET-*T7*-4Z8G4I1T-argW (hereafter called 4Z8G4I1T) constructs.

All of the primers and constructs used in this study are listed in Tables S1 and S2.

### Reporter gene analysis

Fluorescent signals were detected by confocal microscopy (Leica TCS SP5). The strains (*E. coli* BL21 (DE3)) containing the target plasmids were grown in LB at 37 °C and 180 rpm in shaking flask cultures. For the *luc* genes constructed under the *T7* promoter, isopropyl β-D-1-thiogalactopyranoside (IPTG, 0.5 mM) was added into the medium when the bacteria grew to OD_600_ = 0.3. The cells were then collected (in three parallel groups for each strain with 5.0 mL per sample) after 0, 0.5, 1.0, 2.0, 3.0, 5.0, and (occasionally) 7.0 h. The cells were collected after centrifugation at 5000 rpm for 3 min, and re-suspended in 2 mL of 0.85% NaCl solution. The re-suspended cells were lysed by sonication on ice for a total time of 3 min using 50% probe amplitude and the settings were: sonic ON 3 sec, OFF 3 sec. The luciferase activity was assayed using the luciferase Reporter Gene Assay Kit (Beyotime, China), and the expression level of luciferase was reported in Relative Light Units (RLUs). We repeated the growth of the cell and the analysis of the reporter genes for three times. Under each test system (pET-*T7T7*-*n*AGGluc/pACYC-trc-argW, pET-*T7*-*n*AGGluc/pACYC-trc-argW, pET-*T7*-*n*AGGluc-argW), RLUs at rare codon numbers^−1^ (n^−1^) were fitted to the equation:


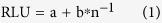


Eq (1) is a linear function where the luciferase activity obtained from insertion of the rare codon (RLU) as the function of rare codon numbers^−1^ (n^−1^), where a and b is constants; “a” is the intercept of the function and “b” is the slope of the function.

### Culture conditions for fatty acid biosynthesis

Normal growth condition was followed to profile fatty acids from WT and engineered strain of *E.coli*. Single colonies of *E. coli* ZGIT, 4Z4G4I4T, 4Z8G4I1T and WT were inoculated in 5 mL of LB medium supplemented with 50 μg·mL^−1^ kanamycin and cultured overnight at 37 °C. Three percent (v/v) inoculations were added aseptically to a 250 mL flask containing 50 mL of LB medium supplemented with 15 g·L^−1^ glucose and 50 μg·mL^−1^ kanamycin and then cultivated at 37 °C under 150 rpm. The cultures were induced by addition of 0.5 mM IPTG at OD_600_ = 0.6, and samples were collected 20 h post-induction for fatty acid analysis.

### Free fatty acid extraction and analysis

Twenty-milliliter samples of cell culture (three replicates for each sample) were centrifuged at 8000 rpm for 10 min to separate cell-associated fatty acids from extracellular fatty acids. Fatty acid extraction was carried out as described earlier^34^. The fatty acids extracted from the supernatant were analyzed by GC/MS using a 5975 C Series MSD and Agilent 6850 equipped with an HP-5 MS column (30 m × 0.32 mm, film thickness of 0.25 mm). Helium was used as a carrier gas. The temperatures of the injector and detector were 250 °C and 280 °C respectively. The GC elution conditions were as follows: 100 °C as the starting temperature (for 5 min), 15 min ramp to 250 °C, and 5 min holding at 250 °C. All samples were spiked with pentadecanoic fatty acid (C15) as an internal standard. We repeated the growth of the cell and the analysis of the fatty acid products for three times.

## Additional Information

**How to cite this article**: Wang, Y. *et al.* An Engineered Rare Codon Device for Optimization of Metabolic Pathways. *Sci. Rep.* **6**, 20608; doi: 10.1038/srep20608 (2016).

## Supplementary Material

Supplementary Information

## Figures and Tables

**Table 1:**
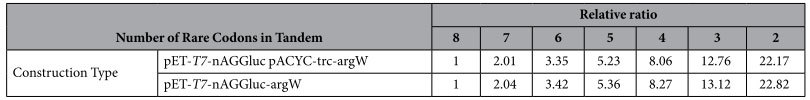
Relative ratio of luciferase activity obtained by different types of constructions.

The relative activity ratio is defined by (RLU obtained by nAGGluc)/(RLU obtained by 8AGGluc). The ratio of 3 AGGs, 5 AGGs or 7 AGGs is predicated by using Eq. (1).
