# Supplementary material for: An Engineered Rare Codon Device for Optimization of Metabolic Pathways
Source: Sci Rep. 2016 Feb 8;6:20608. doi: 10.1038/srep20608 (PMC4745014; doi:10.1038/srep20608)
Supplement: Supplementary Information [file srep20608-s1.pdf]

**An Engineered Rare Codon Device for Optimization of Metabolic Pathways**

You Wang[1,2#], Chunying Li[1,2#], Md. Rezaul Islam Khan[1#], Yushu Wang[1], Yunfeng Ruan[1,2], Bin Zhao[1,2], Bo Zhang[1,2], Xiaopan Ma[1,2], Kaisi Zhang[1,2], Xiwen Zhao[1,2], Guanhao Ye[1,2], Xizhi Guo[1], Guoyin Feng[1], Lin He[1,2,3]*, Gang Ma[1,2]*

1 Bio-X institutes, Key Laboratory for the Genetics of Developmental and Neuropsychiatric Disorders (Ministry of Education), Shanghai Jiao Tong University, Shanghai 200240, PR China

2 2011 SJTU-BioX-Shanghai Team for The International Genetically Engineered Machine Competition (iGEM), Shanghai Jiao Tong University, Shanghai, PR China

3 Shanghai genomePilot Institutes for Genomics and Human Health, Shanghai, 200030, China

# These authors contributed equally to this work.

* Corresponding authors.

Gang Ma: magang2004@gmail.com, Fax: 86-21-34207232

Lin He: helinhelin@gmail.com, Fax: 86-21-62932059

**Table S1. Primers used in this study**

| **Primer name** | **sequence (5' to 3')** |
|---|---|
| **2luc-NcoI-F** | **CCCCCCATGG**CGAGGAGGGCGGCGGAAGACGCCAAAAACATAAAG |
| **4luc-NcoI-F** | **CCCCCCATGG**CGAGGAGGAGGAGGGCGGCGGAAGACGCCAAAAACATAAAG |
| **6luc-NcoI-F** | **CCCCCCATGG**CGAGGAGGAGGAGGAGGAGGGCGGCGGAAGACGCCAAAAACATAAAG |
| **8luc-NcoI-F** | **CCCCCCATGG**CGAGGAGGAGGAGGAGGAGGAGGAGGGCGGCGGAAGACGCCAAAAACATAAAG |
| **luc-NdeI-R** | **CCCCCCATATG**TTTACACGGCGATCTTTCC |
| **rfp - F** | **GGAATTCCATATG**GCTTCCTCCGAAGACG |
| **rfp - R** | **CCGCTCGAG**TTAAGCACCGGTGGAGTGAC |
| **6rfp-F** | **GGAATTCCATATG**AGGAGGAGGAGGAGGAGGGCTTCCTCCGAAGACG |
| **ampR-F** | CCTGTCGCTTGCGGTATTCGGCGGCATCCGCTTACAGACA |
| **ampR-R** | CAGTGAGCGAGGAAGCGGAACGCTCAGTGGAACGAAAA |
| **2blaluc-F** | AATGCTTCAATAATATTGAAAAAGGAAGAGTATGAGGAGGAGGGAAGACGCCAAAAAC |
| **4blaluc-F** | AATGCTTCAATAATATTGAAAAAGGAAGAGTATGAGGAGGAGGAGGAGGGAAGACGCCAAAAAC |
| **6blaluc-F** | AATGCTTCAATAATATTGAAAAAGGAAGAGTATGAGGAGGAGGAGGAGGAGGGAAGACGCCAAAAAC |
| **8blaluc-F** | AATGCTTCAATAATATTGAAAAAGGAAGAGTATGAGGAGGAGGAGGAGGAGGAGGAGGGAAGACGCCAAAAAC |
| **LucAmp-R** | ACGGGAGGGCTTACCATCTGGTTACACGGCGATCTTTCC |
| **trc-F** | CCACCATACCCACGCCGAAACAAACAAAAAGAGTTTGTAGAAACGC |
| **trc-R** | TTGCCGTTACGCACCACCCGGTATTTTCTCCTTACGCATCTGT |
| **argW-F** | TTGTGAGCGGATAACAATTTCACACGTCCTCTTAGTTAAATGGATATAACGAG |
| **argW-R** | GTGGCAGCAGCCTAGGTTAA*G*TCAACGATCTGAAGCGAACCAT |
| **aspV - F** | CCACCATACCCACGCCGAAACCTAAAAATAGCGACTTGGGC |
| **aspV - R** | TTGCCGTTACGCACCACCCTTGAACAGAGAAATGGTGGAA |
| **T7-F** | AGAATGAATCACCGATACGCAGATCTCGATCCCGCGAA |
| **T7-R** | CCTACGAGTTGCATGATAAAGACAAAAAACCCCTCAAGACCC |
| **aspV-CCU-F** | AGTCGGTTAGAATACCTGCCT***CCT***ACGCAGGGGGTCGC |
| **aspV-CCU-R** | GCGACCCCCTGCGT***AGG***AGGCAGGTATTCTAACCGACT |
| **TAGluc-NcoI-F** | **CCCCCCATGG**CGTAGAGGAGGGCGGCGGAAGACGCCAAAAACATAAAG |
| **TDRS-F** | **CATGCC ATGG**GCGTTCTGCCGCTTGACTCT |
| **TDRS-R** | **GGAATTCCATATG**TCAGTTATTCTCAGCCTTCTTCACA |
| **FB-argW-F** | **GGAATTCCATATG**GAAAAATTATAAAAACCCG |
| **FB-argW-R** | **CCGCTCGAG**GTCAACGATCTGAAGCG |

Note:

| | |
|---|---|
| underlined letters | the homologous sequence used for overlap extension PCR |
| **blod letters** | the restriction enzyme sites |
| ***italic and blod letters*** | the mutation points |

**Table S2. Vectors used in this study**

| Vector name | Description |
|---|---|
| **pET-T7-2AGGluc** | lucferase gene with 2 AGG codons inserted after the second codon was inserted into pET28a between *Nco*I and *Nde*I under the T7 promoter |
| **pET-T7-4AGGluc** | lucferase gene with 4 AGG codons inserted after the second codon was inserted into pET28a between *Nco*I and *Nde*I under the T7 promoter |
| **pET-T7-6AGGluc** | lucferase gene with 6 AGG codons inserted after the second codon was inserted into pET28a between *Nco*I and *Nde*I under the T7 promoter |
| **pET-T7-8AGGluc** | lucferase gene with 8 AGG codons inserted after the second codon was inserted into pET28a between *Nco*I and *Nde*I under the T7 promoter |
| **pET-ampR** | *amp*R operon from pUC18 was amplified and inserted into pET28a replacing a nonessential region |
| **pET-bla-4AGGluc** | the ORF of luciferase gene inserted 4 AGG codons after initial codon replacing the ORF of *amp*R gene on pET-bla |
| **pET-bla-6AGGluc** | the ORF of luciferase gene inserted 6 AGG codons after initial codon replacing the ORF of a*mp*R gene on pET-bla |
| **pET-bla-8AGGluc** | the ORF of luciferase gene inserted 8 AGG codons after initial codon replacing the ORF of *amp*R gene on pET-bla |
| **pET-T7-RFP** | wild type RFP gene was inserted into pET28a between *Nde*I and *Xho*I under the T7 promoter |
| **pET-T7-6AGGRFP** | RFP gene with 6 AGG codons inserted after initial codon was inserted into pET28a between *Nde*I and *Xho*I under the T7 promoter |
| **pACYC-trc** | *lac*I regulator, trc promoter and *rrn*B ternimator were amplified together from pTrc99b and inserted into pACYC184 replacing *Cm*R operon |
| **pACYC-trc-argW** | insert *arg*W after the trc promoter on pACYC-trc |
| **pACYC-aspV** | *asp*V operon was amplified from the genome of *E.coli* and inserted into pACYC184 replacing the *Cm*R operon |
| **pACYC-T7-aspV** | T7 promoter from pET28a was inserted into pACYC-aspV replacing a fragment of blank sequence |
| **pACYC-T7-aspV(CCU)** | mutated the anticodon part of *asp*V gene on pACYC-T7-aspV from GTC to CCT |
| **pACYC-T7-4AGGluc-aspV(CCU)** | luciferase gene with 4 AGG codons inserted after initial codon was inserted after T7 promoter on pACYC-T7-aspV(CCU) |
| **pACYC-T7-6AGGRFP-aspV(CCU)** | RFP gene with 6 AGG codons inserted after initial codon was inserted after T7 promoter on pACYC-T7-aspV(CCU) |
| **pET-T7-TDRS** | the truncated aspRS was amplified from the genome of *E.coli* and inserted after T7 promoter on pET28a using overlap extension PCR |
| **pET-T7-2AGGluc-argW** | *arg*W gene was amplified from *E.coli* genome and inserted after 2AGG-luc gene before T7 ternimator on pET-T7-2AGGluc |
| **pET-T7-4AGGluc-argW** | *arg*W gene was amplified from *E.coli* genome and inserted after 4AGG-luc gene before T7 ternimator on pET-T7-4AGGluc |
| **pET-T7-6AGGluc-argW** | *arg*W gene was amplified from *E.coli* genome and inserted after 6AGG-luc gene before T7 ternimator on pET-T7-6AGGluc |

| | |
|---|---|
| **pET-T7-8AGGluc-argW** | *arg*W gene was amplified from *E.coli* genome and inserted after 8AGG-luc gene before T7 ternimator on pET-T7-8AGGluc |

**Supplementary Figure legends**

**Fig. S1: Fatty acid metabolism pathway in *E. coli.*** FabA, FabB, FabD, FabF, FabG, FabH, FabI, FabZ are the main enzymes of the fatty acid biosynthesis pathway in *E.coli.* FabB, FabF, Fab G, FabI, FabZ/FabA are mainly responsible for fatty acid elongation. TesA is the enzyme that is able to release free fatty acid by hydrolysis of acyl-ACP species. FabG, FabZ, FabI and TesA were picked as targets for manipulation in these experiments based on previous studies.

**Fig. S2: RFP expression controlled by rare codon devices.** (**A**) Constructs for testing rare codon devices. RFP containing 6 AGGs and $tRNA^{Arg}$ (CCU) were expressed under separate *T7* promoters (blue). (**B**) Cells containing *rfp* without the rare AGG codon and $tRNA^{Arg}$ (CCU) expressed RFP (upper row). RFP expression nearly ceased upon insertion of 6 AGG codons (S2A) (middle row). RFP expression was restored in cells containing pACYC-trc-argW (coded for $tRNA^{Arg}$ (CCU)) (bottom row). (**C-I**) Time-course assays of luciferase activity. Black line indicates luciferase activity in the cell carrying pET-T7-nAGGluc and pACYC-trc-argW (C-F, n = 2, 4, 6, 8) or pET-bla-*n*AGGluc and pACYC-trc-argW (G-I, n = 4, 6, 8). Red line indicates luciferase activity in the cell carrying pET-T7-nAGGluc (C-F, n = 2, 4, 6, 8) or pET-bla-*n*AGGluc (G-I, n = 4, 6, 8).

**Fig S3: Protein levels of luciferase with various numbers of rare AGG codon insertions after the start codon. (A)** SDS-PAGE of lysates of cells containing rare codon devices

shown in Fig. 1A. The upper bands (nAGGLuc) represent luciferase with various numbers of arginines at the N terminus. The lower bands are western blots of DnaK protein, which was chosen as the internal control. **(B)** The bands in Fig. S3A were quantified in terms of relative protein amounts using Quantity One® software.

**Fig. S4: Control of luciferase expression via TDRS and mutated tRNA$^{Asp}$ (GUC→CCU).** (**A**) Sketch showing TDRS and mutated tRNA$^{Asp}$ (GUC→CCU). (**B**) Luciferase activity was detected only in the cell carrying plasmids containing *luciferase* with 4 AGG codon insertions, mutated tRNA$^{Asp}$ (GUC→CCU), and TDRS (right-hand column). Only the luciferase substrate (considered as background) was detected from the cell containing *luciferase* with 4 AGG codon insertions and mutated tRNA$^{Asp}$ (GUC→CCU) (left-hand column).

**Fig. S5: Control of kanamycin resistance gene expression using truncated MetRS and tRNA$^{fMet}$ (CAT→TCG).** (**A**) Sketch of the test system. The start codon of *KanR* was mutated to CGA to permit recognition by tRNA$^{fMet}$ (CAT→TCG). The tRNA$^{fMet}$ (CAT→TCG) was charged using truncated MetRS. (**B**) Expression of *KanR* (ATG→CGA) was controlled by truncated MetRS and tRNA$^{fMet}$ (CAT→TCG). The left-hand figure shows a cell carrying truncated MetRS, tRNA$^{fMet}$ (CAT→TCG), and *KanR* (ATG→CGA) grown on a plate containing kanamycin. The cell that carried only tRNA$^{fMet}$ (CAT→TCG) and *KanR* (ATG→CGA) could not grow on the kanamycin plate showed in the right-hand figure. "N" and "M" indicate two independent clones.

**Fig. S6: Component analysis of fatty acid products by GC-MS.** Data for the strains **(A**) ZGIT, **(B)** 4Z4G4I4T, and **(C)** 4Z8G4I1T. The X-axis and Y-axis represent retention time and relative amounts, respectively.

**Fig. S7: Analysis of the structures of aminoacyl-tRNA synthetases complexed with cognate tRNAs.** Black arrows indicate the anticodon recognition domains of various aminoacyl-tRNA synthetases. Figures on the left depict natural aminoacyl-tRNA synthetases complexed with cognate tRNAs, while those on the right represent hypothesized truncated aminoacyl-tRNA synthetases without the anticodon recognition domain. (**A**) Arginyl-tRNA synthetase, (**B**) cysteinyl-tRNA synthetase, (**C**) glutaminyl-tRNA synthetase, (**D**) glutamyl-trna synthetase, (**E**) threonyl-tRNA synthetase, (**F**) tryptophanyl-tRNA, (**G**) tyrosyl-tRNA synthetase, and (**H**) methionyl-tRNA synthetase.

Fig. S1

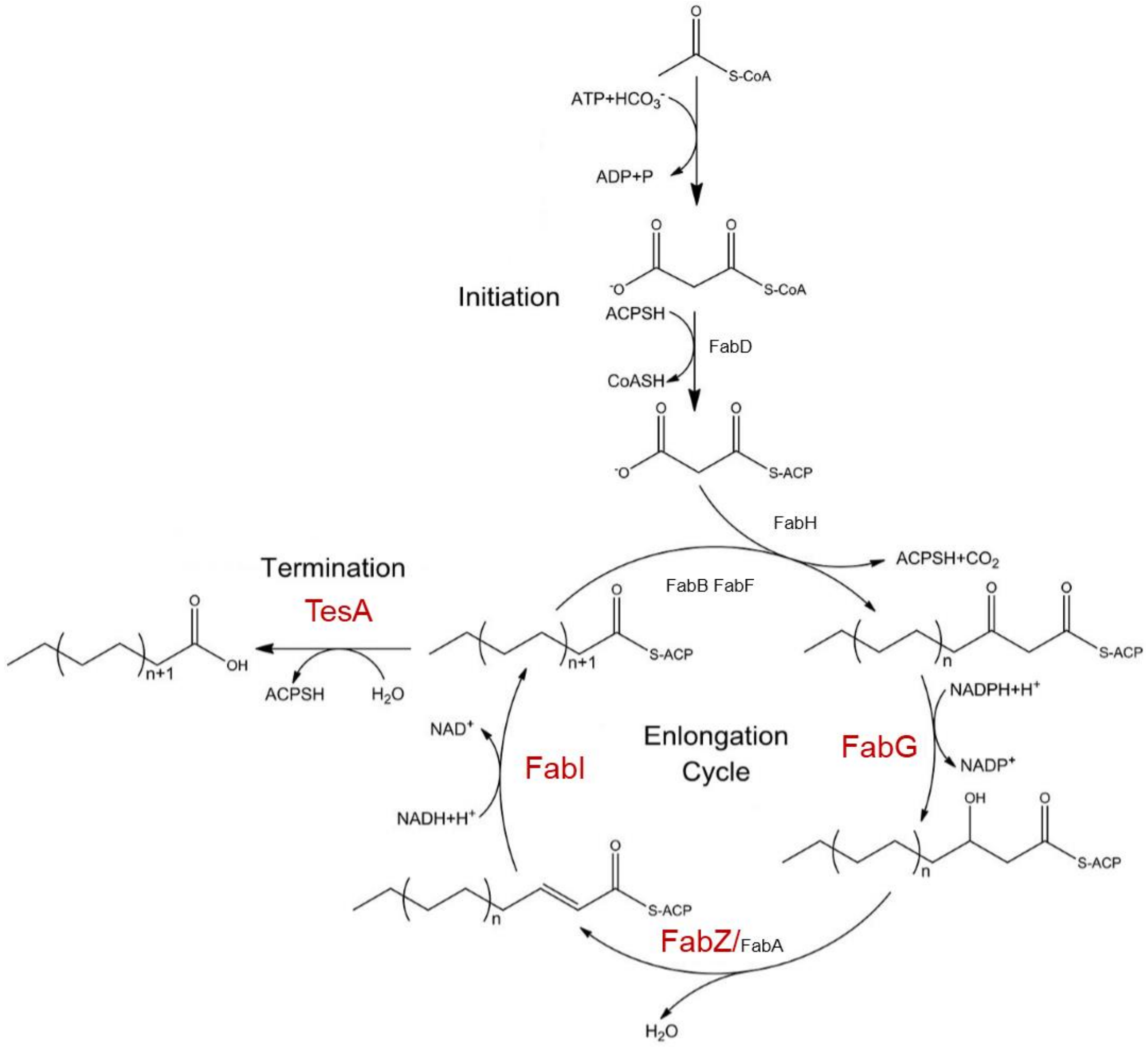
S-CoA
ATP+HCO3-
ADP+P
Initiation
S-CoA
ACPSH
FabD
CoASH
S-ACP
FabH
ACPSH+CO2
FabB FabF
Termination
TesA
OH
n+1
ACPSH
H2O
S-ACP
n
NADPH+H+
NAD+
Enlongation
Cycle
FabG
FabI
NADP+
NADH+H+
OH
S-ACP
FabZ/FabA
H2O

Fig. S2A

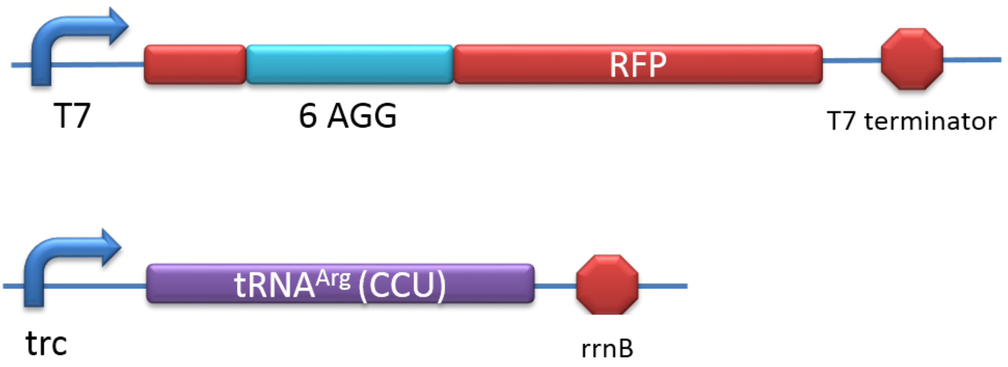


Fig. S2B

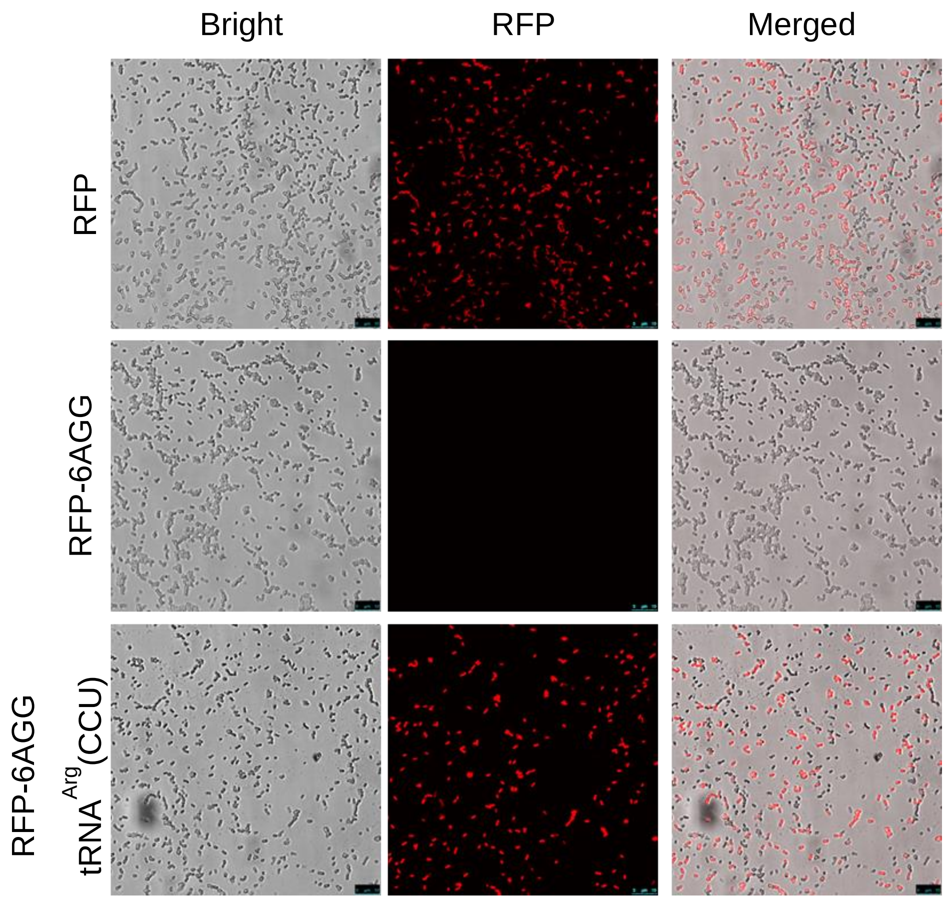

Fig. S2C

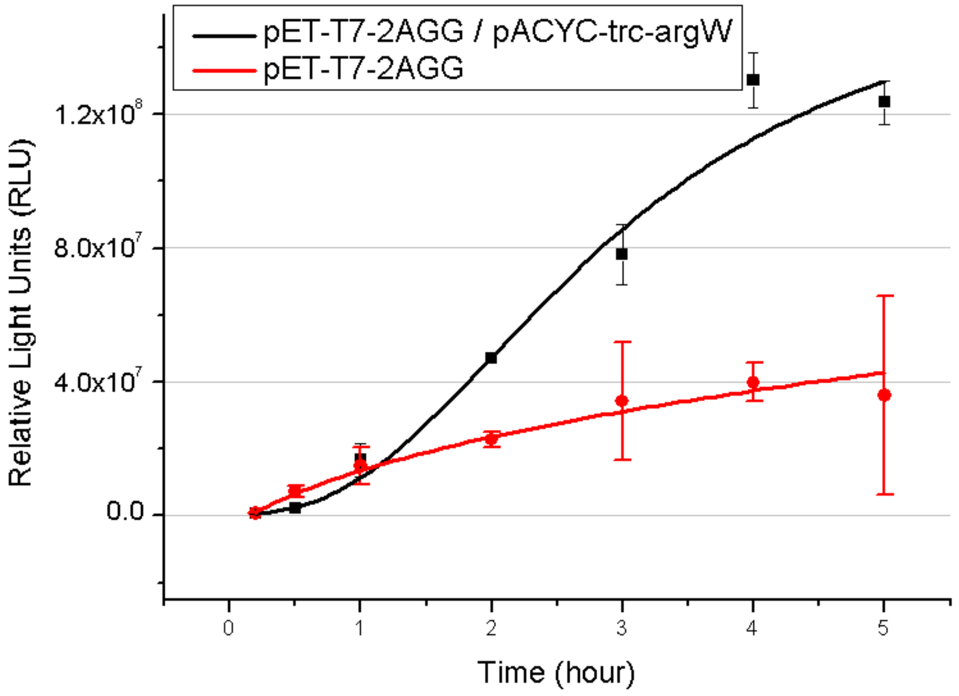


Fig. S2D

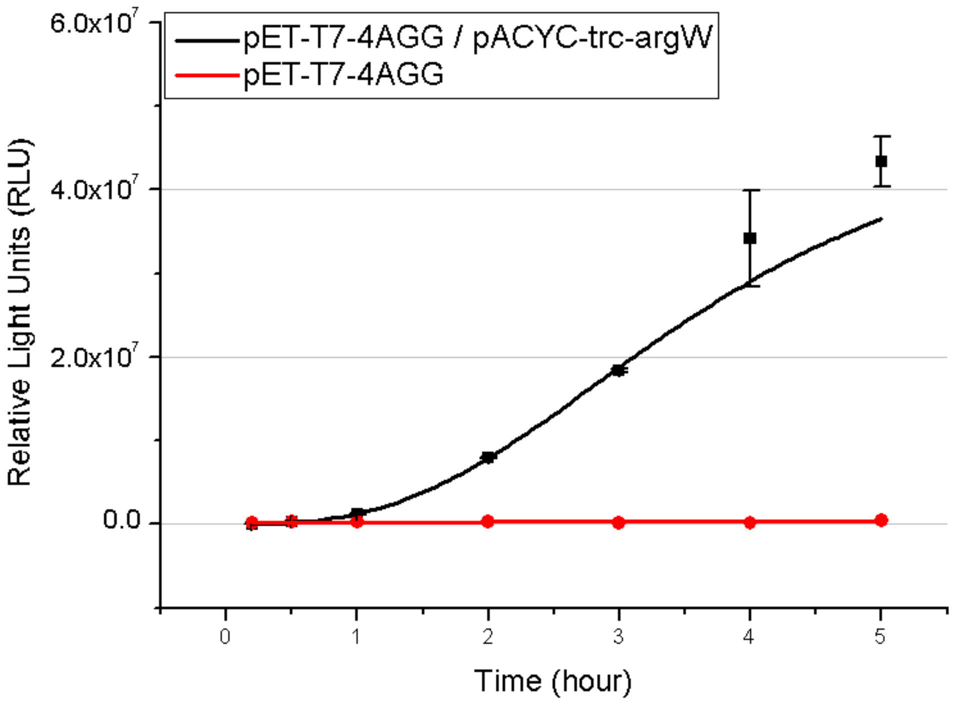

Fig. S2E

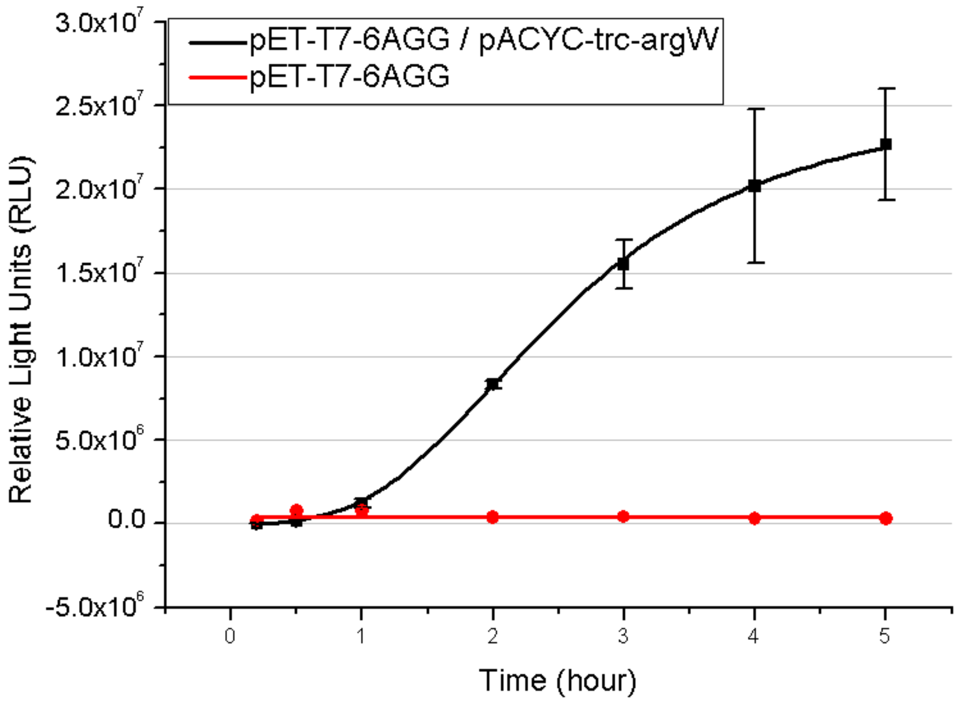


Fig. S2F

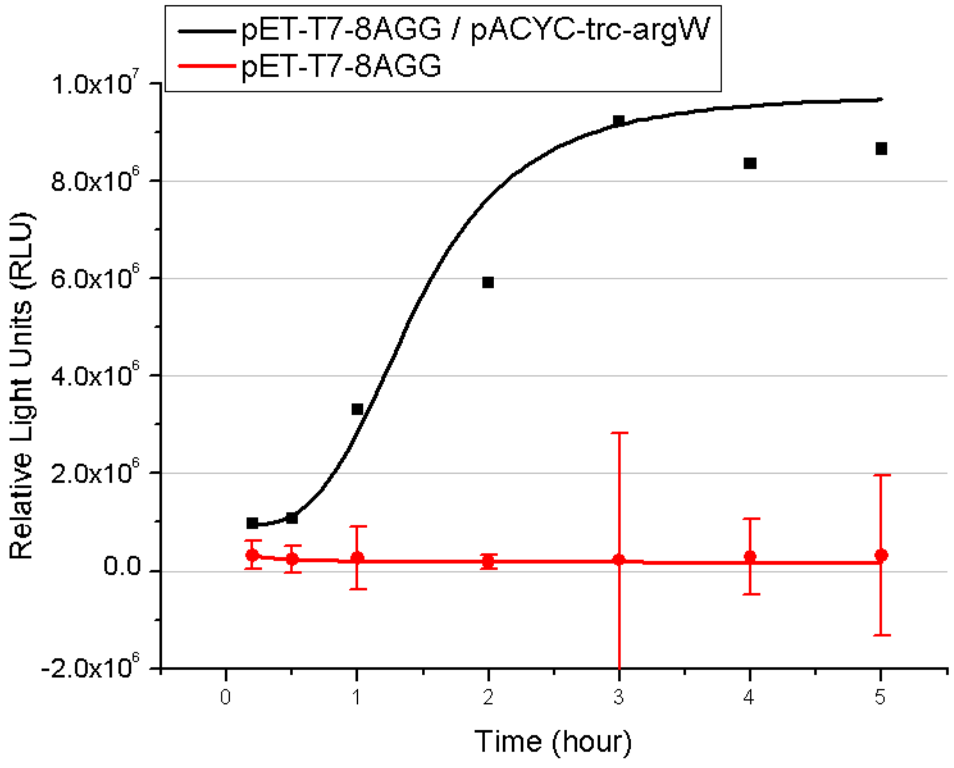

Fig. S2G

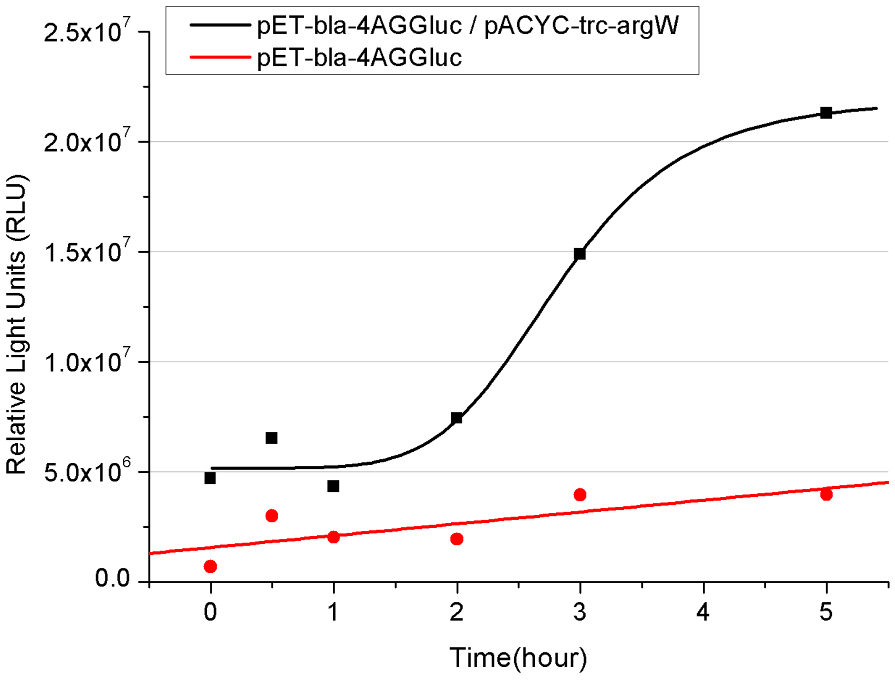


Fig. S2H

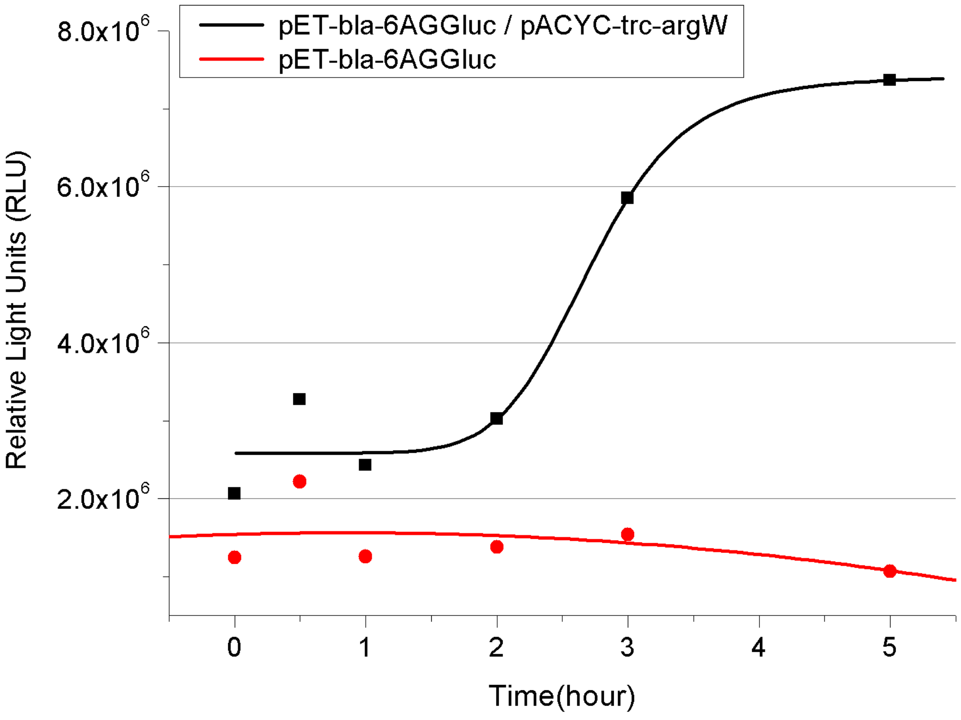

Fig. S2I

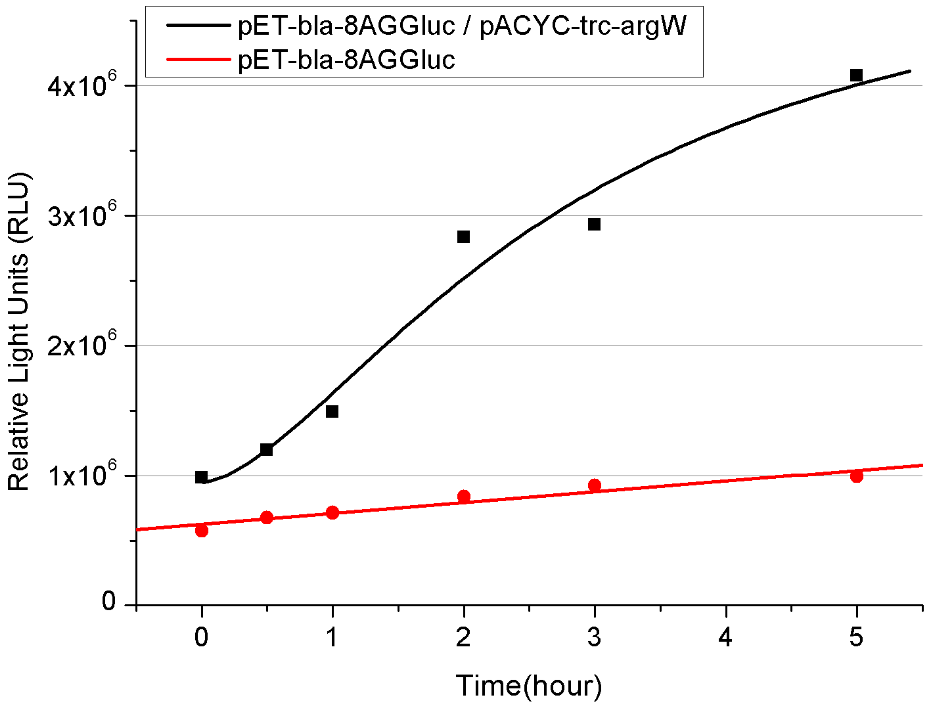

pET-bla-8AGGluc / pACYC-trc-argW
pET-bla-8AGGluc
Relative Light Units (RLU)
4x10^6
3x10^6
2x10^6
1x10^6
0
0
1
2
3
4
5
Time(hour)

Fig. S3A

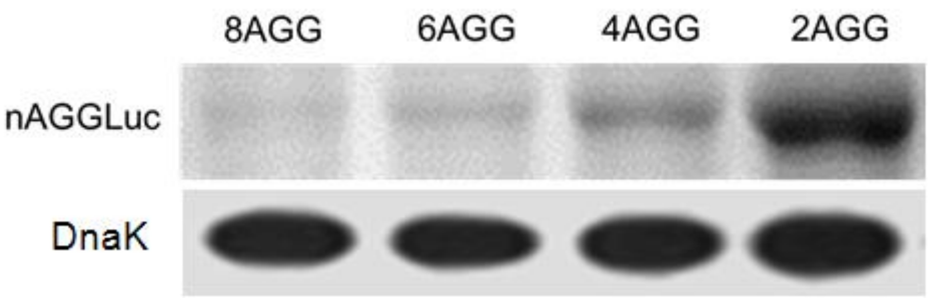


Fig. S3B

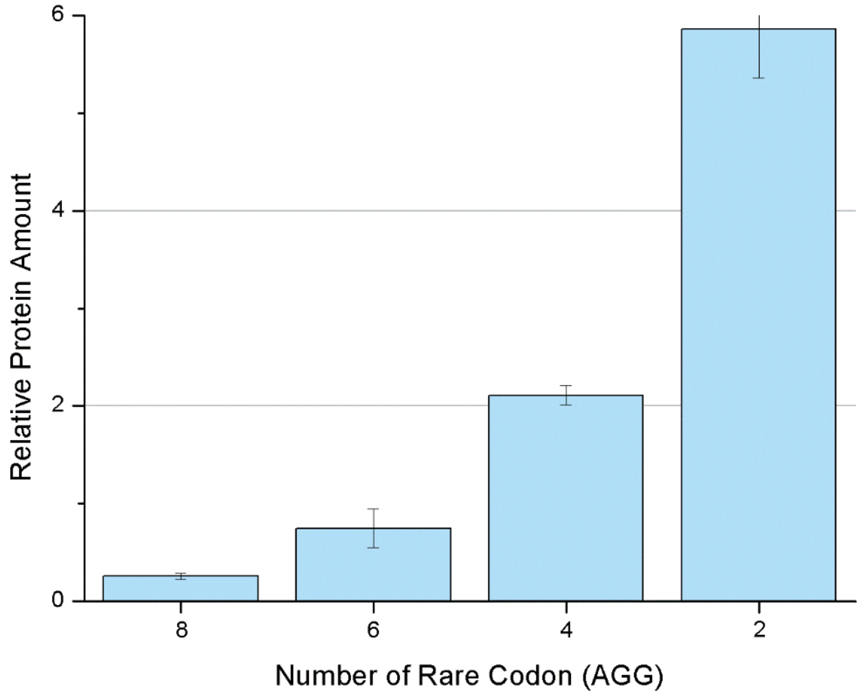

Fig. S4A

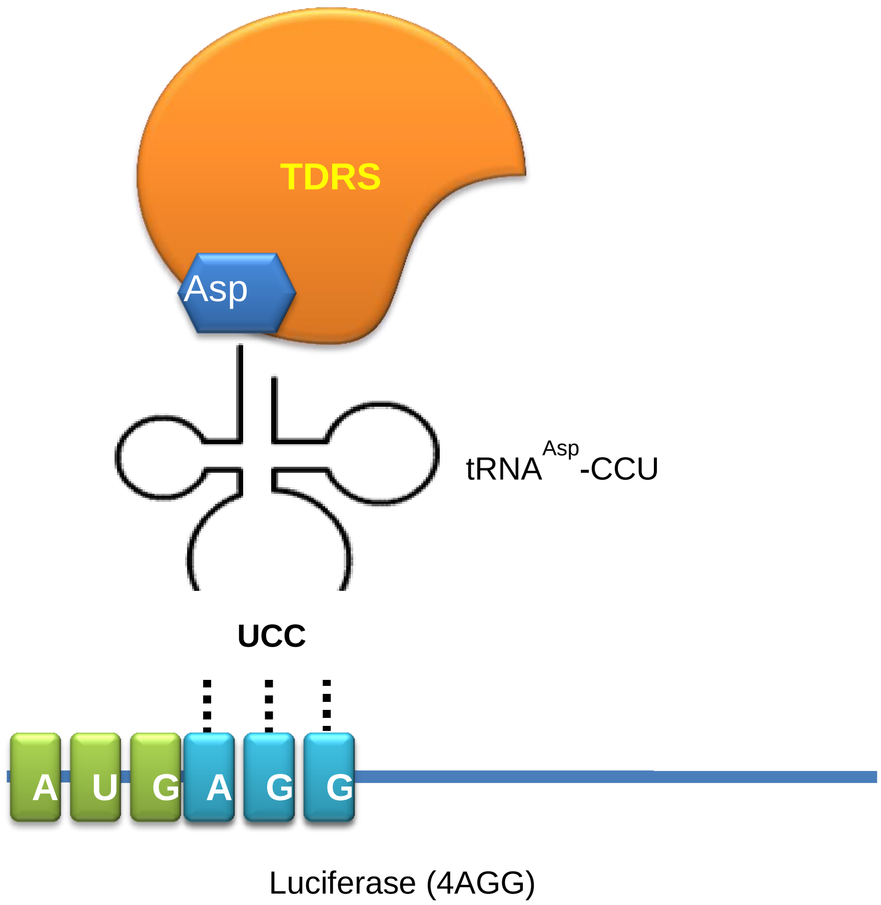


Fig. S4B

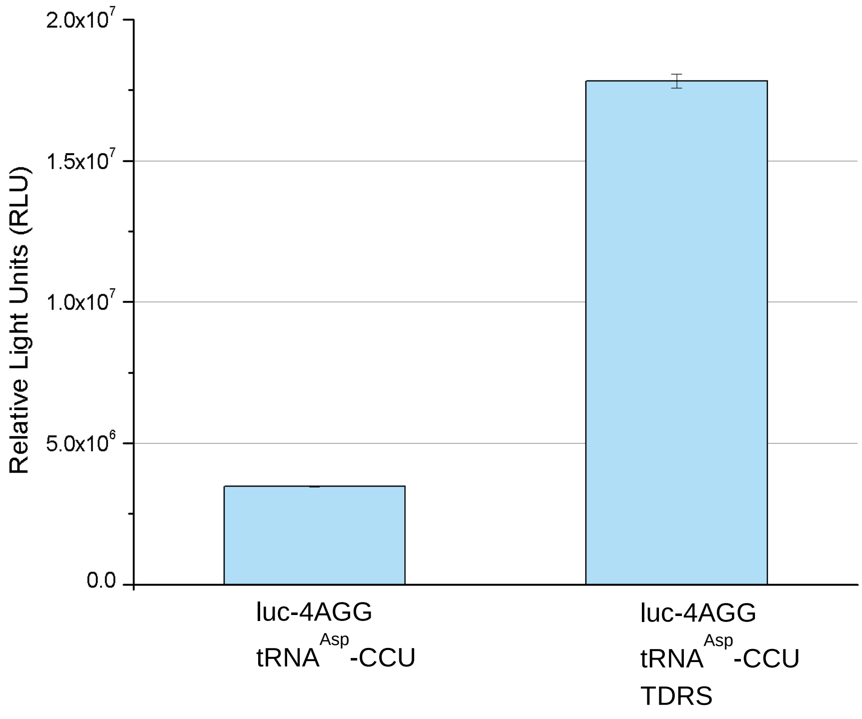

Fig. S5A

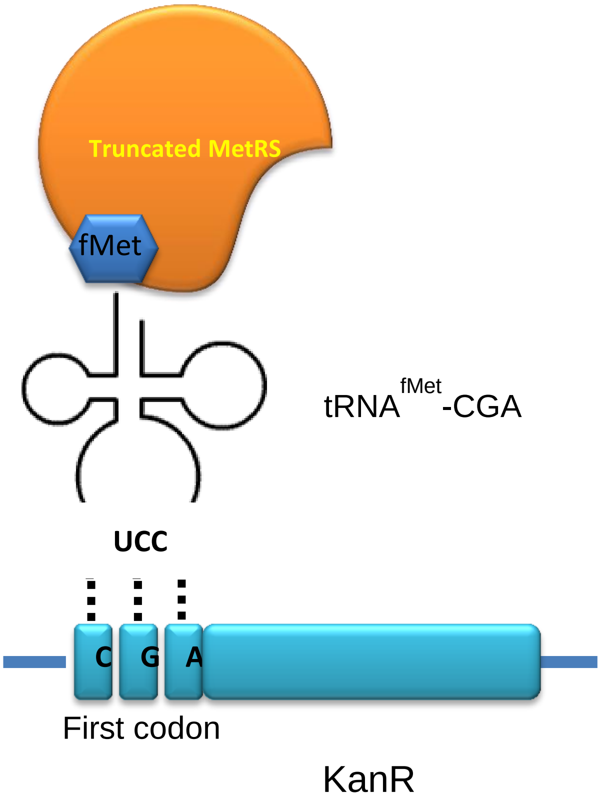


Fig. S5B

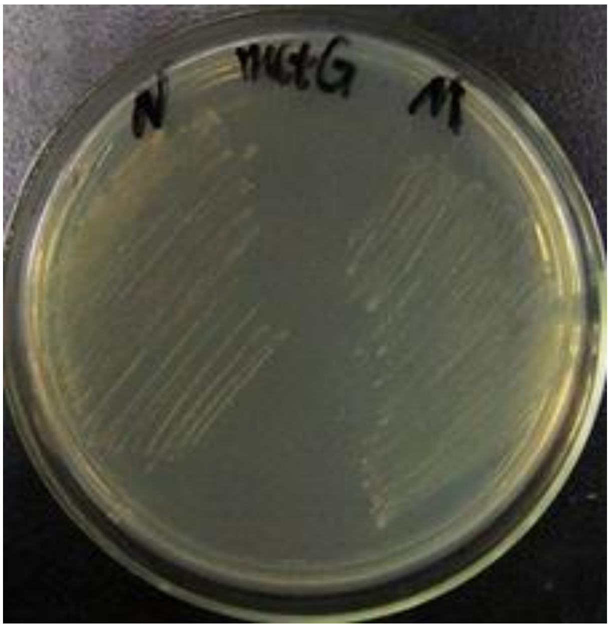

Trucated MetRS

tRNA$^{fMet}$ (CAT→TCG)

KanR (ATG→CGA)

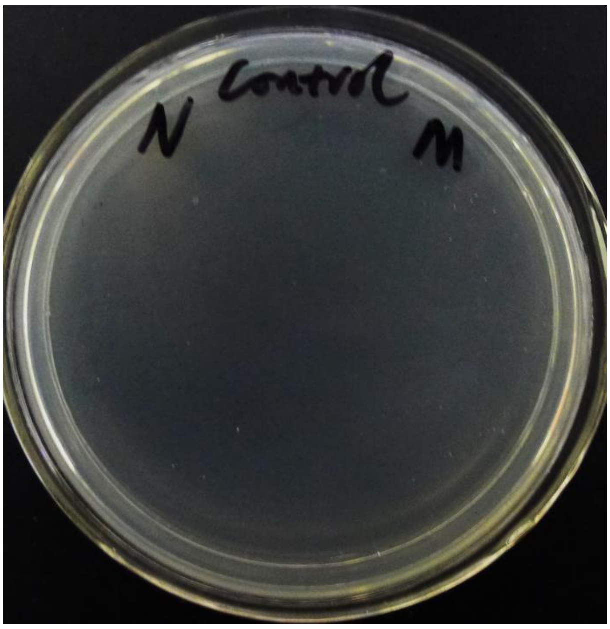

tRNA$^{fMet}$ (CAT→TCG)

KanR (ATG→CGA)

Fig. S6A

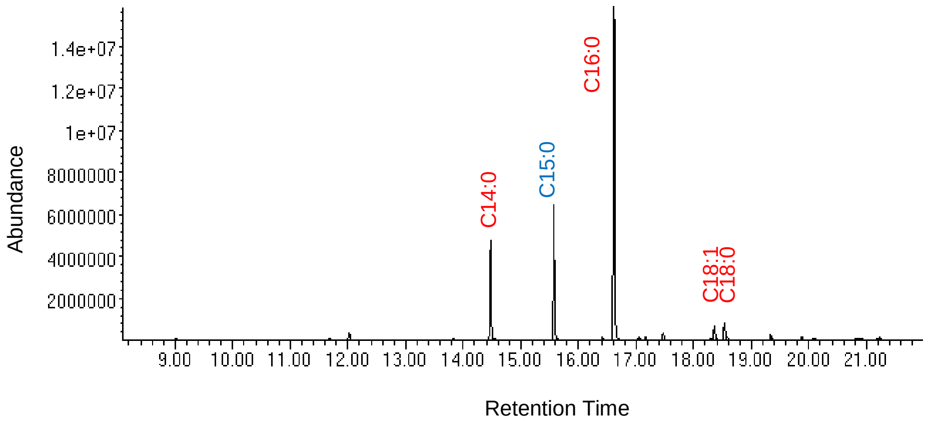


Fig. S6B

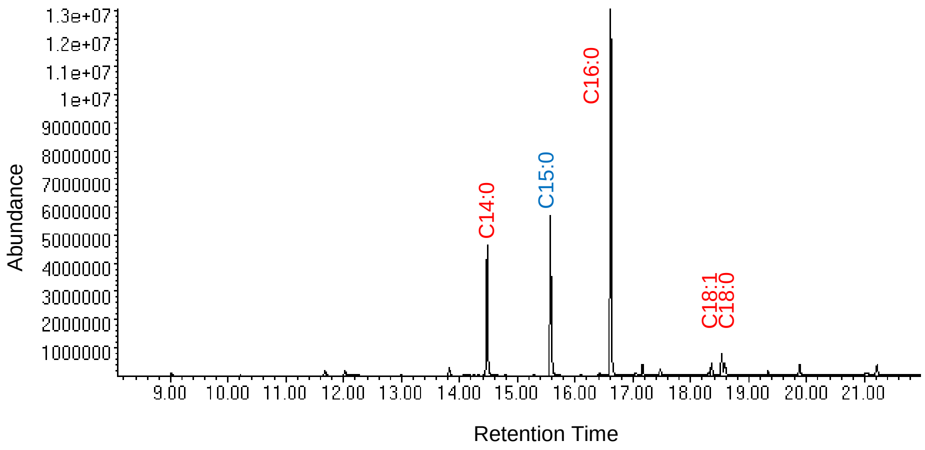


Fig. S6C

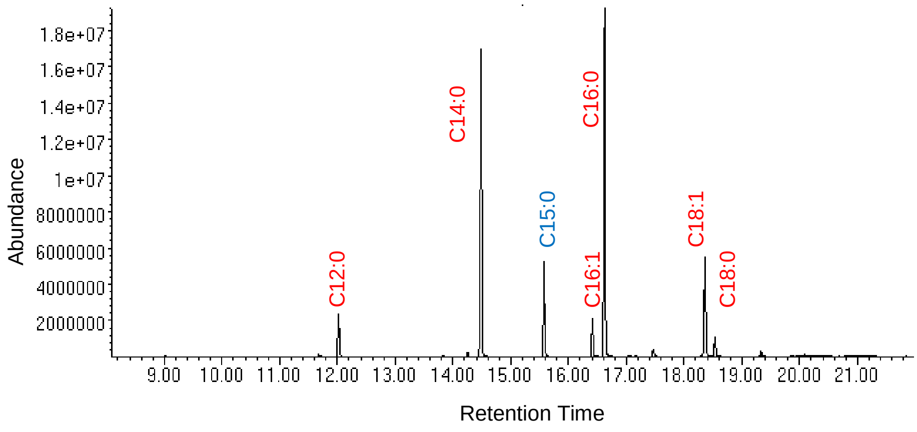

Fig. S7A

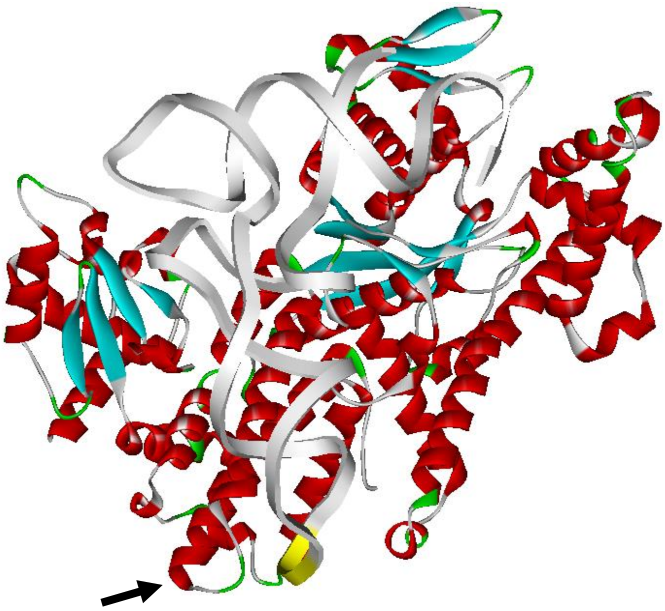

Arginyl-tRNA synthetase

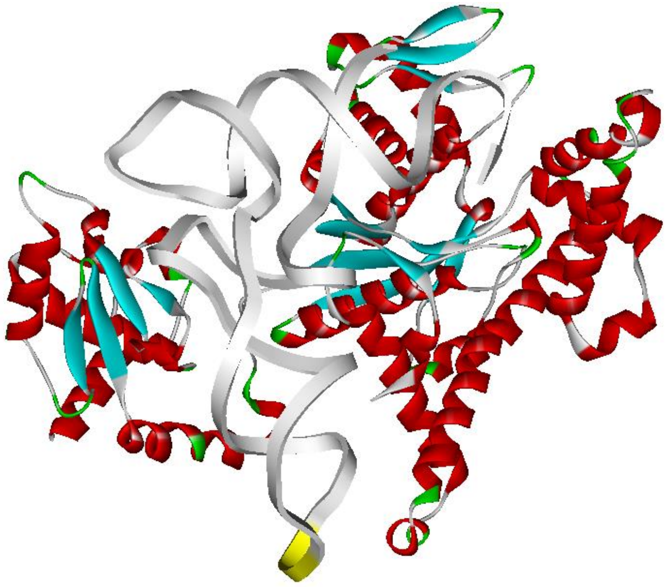

Truncated Arginyl-tRNA synthetase
(anticodon recognition domain deletion (483-607))

Fig. S7B

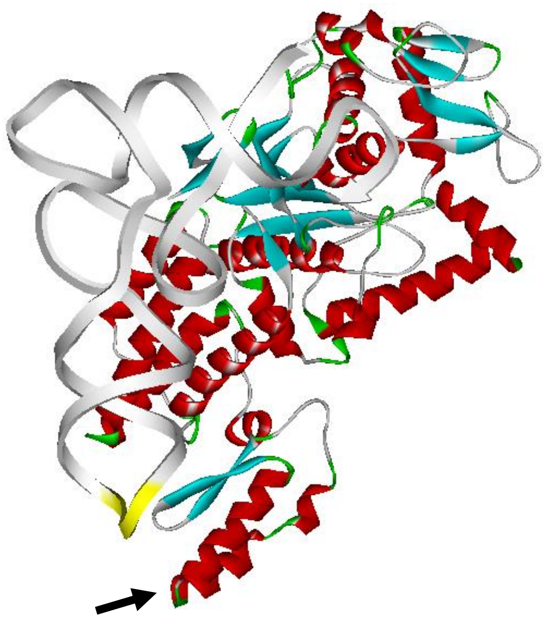

Cysteinyl-tRNA synthetase

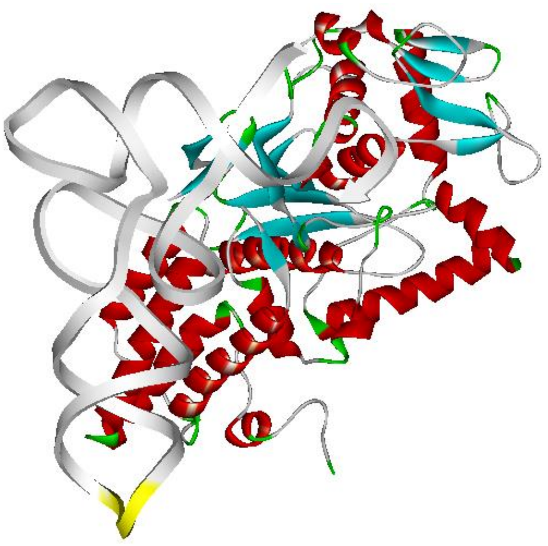

Truncated Cysteinyl-tRNA synthetase
(anticodon recognition domain deletion (413-461))

Fig. S7C

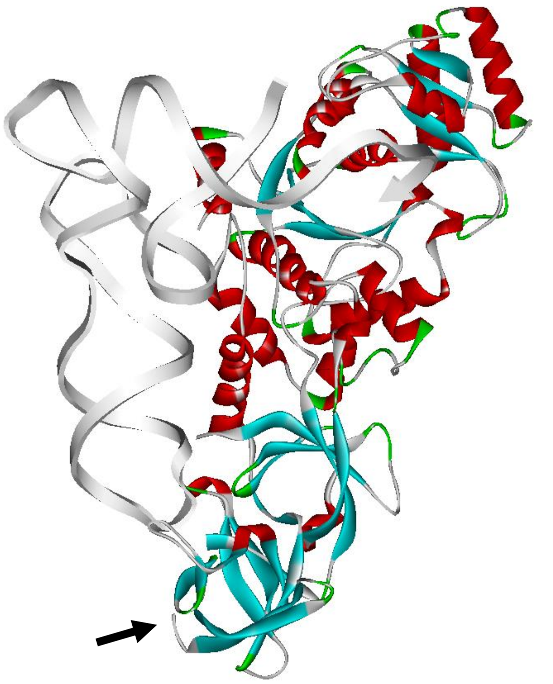

Glutaminyl-tRNA synthetase (PDB 1O0C)

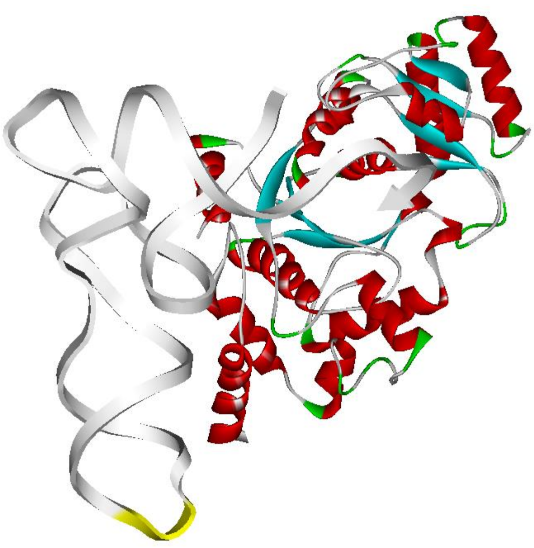

Truncated Glutaminyl-tRNA synthetase
(anticodon recognition domain deletion (340-547))

Fig. S7D

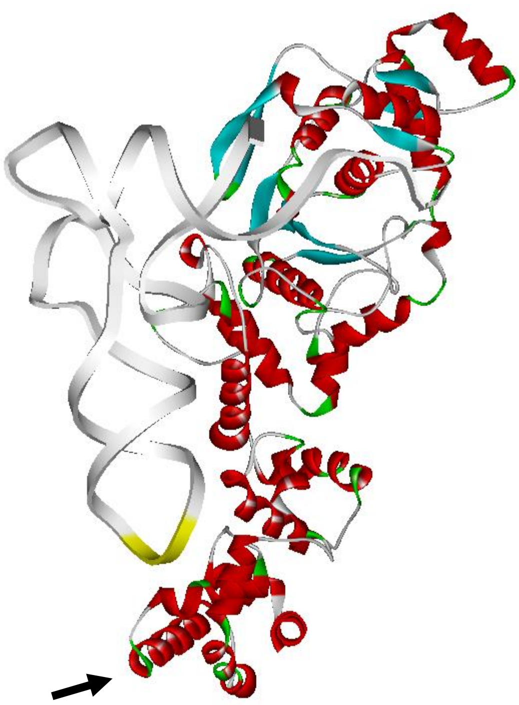

Glutamyl-trna synthetase (PDB 1G59)

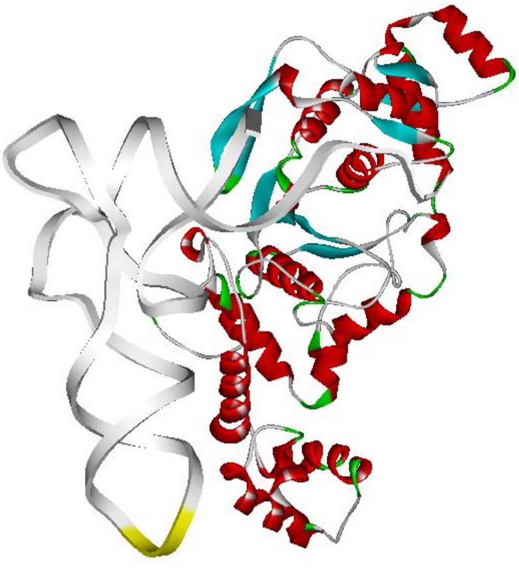

Truncated Glutamyl-trna synthetase
(anticodon recognition domain deletion (375-468))

Fig. S7E

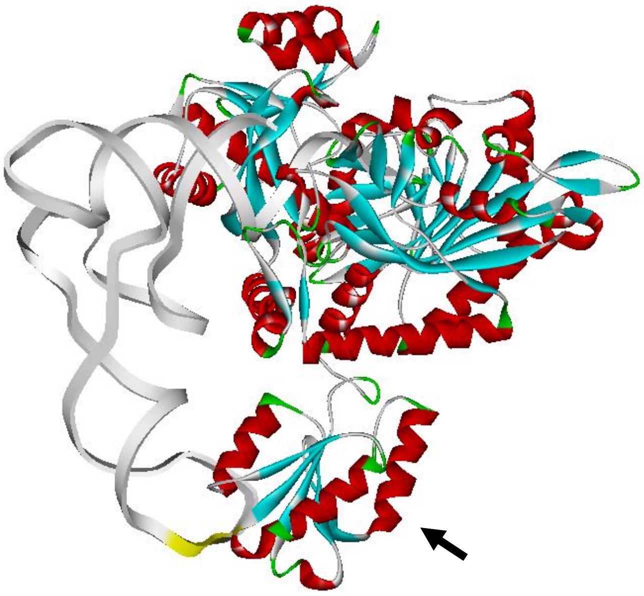

Threonyl-tRNA synthetase (PDB 1QF6)

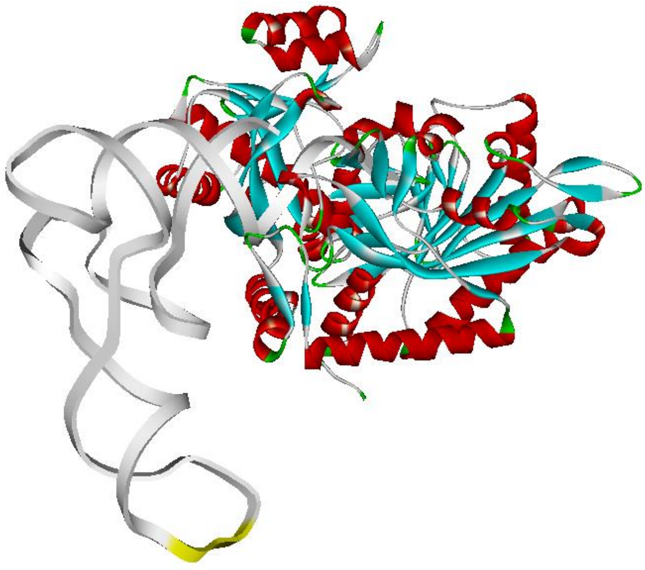

Truncated Threonyl-tRNA synthetase
(anticodon recognition domain deletion (536-642))

Fig. S7F

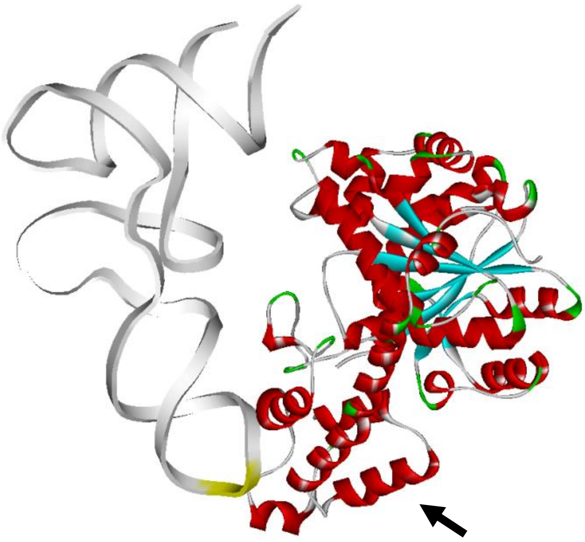

Tryptophanyl-tRNA synthetase (PDB 2AKE)

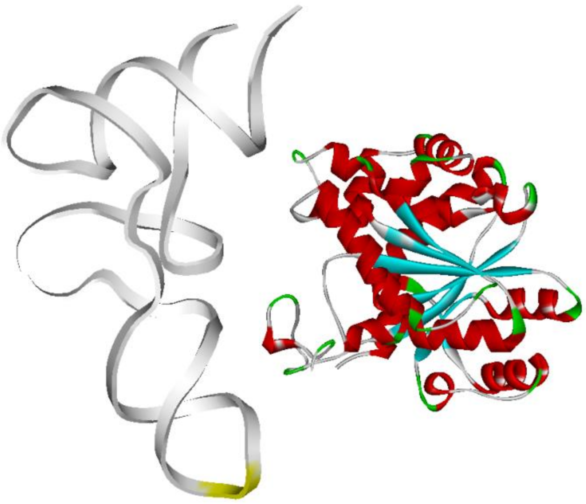

Truncated Tryptophanyl-tRNA synthetase
(anticodon recognition domain deletion (365-469))

Fig. S7G

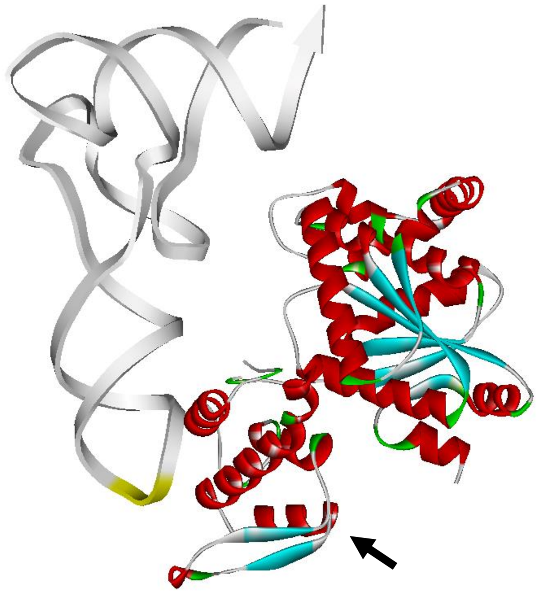

Tyrosyl-tRNA synthetase (PDB 1J1U)

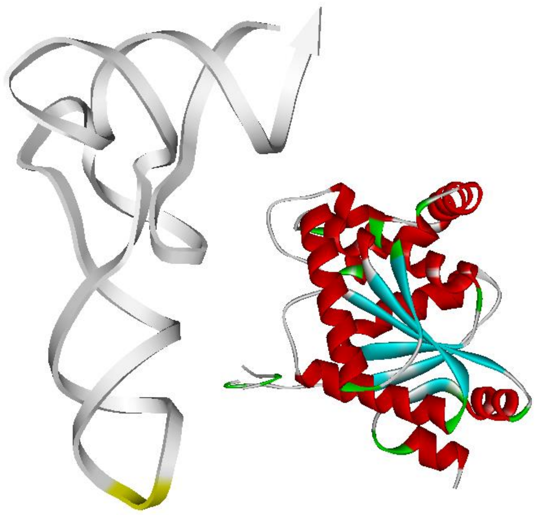

Truncated Tyrosyl-tRNA synthetase
(anticodon recognition domain deletion (219-306))

Fig. S7H

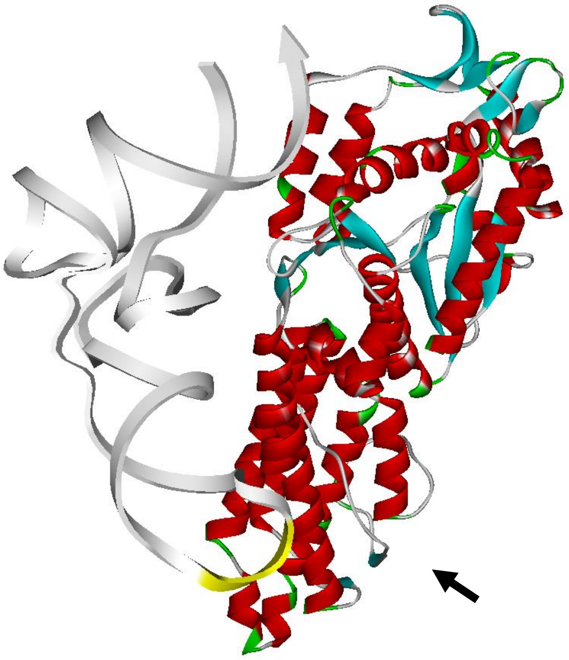

Methionyl-tRNA synthetase (PDB 2CSX)

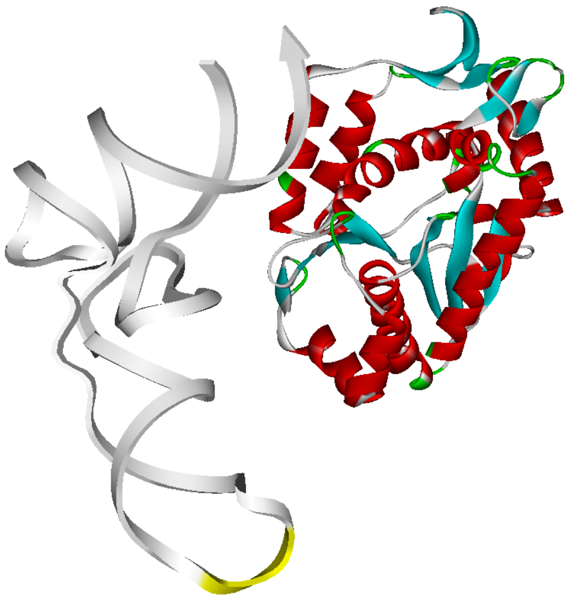

Truncated Methionyl-tRNA synthetase
(anticodon recognition domain deletion (336-497))
